# Robust metabolic transcriptional components in 34,494 patient-derived cancer-related samples and cell lines

**DOI:** 10.1186/s40170-021-00272-7

**Published:** 2021-09-26

**Authors:** V. C. Leeuwenburgh, C. G. Urzúa-Traslaviña, A. Bhattacharya, M. T. C. Walvoort, M. Jalving, S. de Jong, R. S. N. Fehrmann

**Affiliations:** 1 Department of Medical Oncology, Cancer Research Center Groningen, University Medical Center Groningen, University of Groningen, Groningen, The Netherlands; 2 Department of Chemical Biology, Stratingh Institute for Chemistry, University of Groningen, Groningen, The Netherlands

**Keywords:** Transcriptome, Metabolism, Independent component analysis, Tumor microenvironment, Drug sensitivity

## Abstract

**Background:**

Patient-derived bulk expression profiles of cancers can provide insight into the transcriptional changes that underlie reprogrammed metabolism in cancer. These profiles represent the average expression pattern of all heterogeneous tumor and non-tumor cells present in biopsies of tumor lesions. Hence, subtle transcriptional footprints of metabolic processes can be concealed by other biological processes and experimental artifacts. However, consensus independent component analyses (c-ICA) can capture statistically independent transcriptional footprints of both subtle and more pronounced metabolic processes.

**Methods:**

We performed c-ICA with 34,494 bulk expression profiles of patient-derived tumor biopsies, non-cancer tissues, and cell lines. Gene set enrichment analysis with 608 gene sets that describe metabolic processes was performed to identify the transcriptional components enriched for metabolic processes (mTCs). The activity of these mTCs was determined in all samples to create a metabolic transcriptional landscape.

**Results:**

A set of 555 mTCs was identified of which many were robust across different datasets, platforms, and patient-derived tissues and cell lines. We demonstrate how the metabolic transcriptional landscape defined by the activity of these mTCs in samples can be used to explore the associations between the metabolic transcriptome and drug sensitivities, patient outcomes, and the composition of the immune tumor microenvironment.

**Conclusions:**

To facilitate the use of our transcriptional metabolic landscape, we have provided access to all data via a web portal (www.themetaboliclandscapeofcancer.com). We believe this resource will contribute to the formulation of new hypotheses on how to metabolically engage the tumor or its (immune) microenvironment.

**Supplementary Information:**

The online version contains supplementary material available at 10.1186/s40170-021-00272-7.

## Background

Reprogrammed energy metabolism is a hallmark of cancer [1]. Metabolic reprogramming supports the survival, proliferation, and maintenance of cancer cells by ensuring sufficient biosynthetic capacity, redox potential, and energy [2]. Additionally, metabolic reprogramming enables tumor cells to adapt to challenging microenvironmental conditions, such as hypoxia and low nutrient availability, and become resistant to cancer treatment [3]. Moreover, metabolic reprogramming of cancer cells influences the composition and function of immune cells present in the tumor microenvironment (TME), affecting the anti-cancer immune response to immunotherapy [4, 5].

Metabolic dependencies have been successfully exploited to treat cancer, as illustrated by the efficacy of antifolate drugs such as methotrexate [6]. More recent knowledge about cancer cell metabolism has resulted in novel therapeutic targets, such as glutaminase and mutant forms of IDH1/2, currently being evaluated in pre-clinical models and phase I/II clinical trials [7, 8]. However, adverse effects or lack of effectiveness still hamper the clinical development of most metabolic therapies. A potential reason is that many metabolic targeting drugs are developed based on insights derived from model systems of human cancer, which do not fully reflect the complexities of cancer in humans [9]. In particular, cell line models lack the immune cells present in the TME and often require specific metabolic conditions to grow [10, 11].

Evidence is emerging that transcriptional changes play an important role in the metabolic plasticity of cancer cells: gene expression can influence metabolite levels, and metabolic changes can result in altered gene expression [12, 13]. The availability of large numbers of gene expression profiles—from a broad spectrum of cancer types—in the public domain provides a unique opportunity to study metabolic reprogramming in patient-derived cancer tissue.

Almost without exception, these gene expression profiles were generated from complex biopsies that contain tumor cells and cells present in the TME (e.g., immune cells). Accordingly, these profiles represent the average gene expression pattern of all cells present in the biopsy. Therefore, detecting metabolic processes relevant to cancer biology in expression profiles from complex biopsies can be challenging, especially when their transcriptional footprints (TFs) are subtle and concealed by more pronounced TFs from other biological processes or experimental artifacts.

In the present study, we used consensus independent component analysis (c-ICA), a statistical method capable of separating the average gene expression profiles generated from complex biopsies into additive transcriptional components (TCs). This enabled us to detect both the pronounced and more subtle transcriptional footprints of metabolic processes. We performed c-ICA with 32,409 human gene expression profiles obtained from the Gene Expression Omnibus (GEO) and The Cancer Genome Atlas (TCGA), as well as 2085 gene expression profiles obtained from the Cancer Cell Line Encyclopedia (CCLE) and the Genomics of Drug Sensitivity in Cancer (GDSC) portal [14–16]. Comprehensive characterization of the TCs with gene set enrichment analysis (GSEA) identified TCs associated with metabolic processes, i.e., metabolic TCs (mTCs). This enabled us to create a metabolic landscape showing the activity of these mTCs in all 34,494 samples. We demonstrate how this landscape (www.themetaboliclandscapeofcancer.com) can be used to explore the associations between the metabolic transcriptome and drug sensitivities, patient outcomes, and the composition of immune cells in the TME.

## Methods

### Data acquisition, data preprocessing, and c-ICA

A detailed description of the methods is available in the Supplementary Methods. The methods for data acquisition, preprocessing of the four datasets GEO, TCGA, GDSC, and CCLE, and c-ICA were published previously [17].

### Identification of TCs enriched for metabolic processes

Gene sets defining all known types of metabolic processes were selected from gene set collections obtained from the Molecular Signatures Database (MSigDb version 6.1): BioCarta, Gene Ontology – Biological Process (GO-BP), Gene Ontology – Molecular Function (GO-MF), KEGG, and Reactome (see Supplementary Methods for details on selection process). To identify transcriptional components enriched for metabolic processes, gene set enrichment analysis (GSEA) was performed using the selected metabolic gene sets. Enrichment of each metabolic gene set was tested according to the two-sample Welch’s *t*-test for unequal variance between the metabolic set of genes, and Welch’s *t* statistic was transformed to a *Z*-score to allow comparison between gene sets.

To reduce the redundancy in gene sets from different gene set collections, consensus clustering was performed set-wise on the GSEA data for the GEO, TCGA, CCLE, and GDSC datasets. Consensus clustering was performed using the ConsensusClusterPlus-package (v1.51.1) within R, using the default hierarchical clustering algorithm and Pearson correlation distance, a maximum number of clusters (maxK) of 150, 2000 resamplings (reps), with 80% row and 80% column resampling (pFeature and pItem, respectively). The optimal number of clusters (*k*) was determined as the *k* at which the relative change in area under the cumulative distribution function (CDF) curve was minimized (< 0.01). This resulted in a *k* of 50 clusters (Supplementary Fig. 1).

The 50 clusters of gene sets were subsequently used to select transcriptional components based on their enrichment for metabolic processes. Per gene set cluster, the three TCs with the highest absolute enrichment score for any gene set in that cluster were selected. We also selected the three TCs with the highest absolute mean enrichment score for all gene sets in that cluster. These selected TCs were then referred to as metabolic transcriptional components (mTCs).

### Pair-wise gene-level correlations of mTCs between datasets

To correlate two mTCs of different datasets, the subset of genes with an absolute weight higher than 3 in two mTCs was selected. Then, the overlap between these two sets of top genes was determined. Using the gene weights of the overlapping genes in both mTCs, pair-wise correlations were calculated. Specifically, Spearman correlations were performed using the *pspearman*-package (v0.3-0) in R, with a *t*-distribution approximation to determine the *P*-value. As the number of genes with an absolute weight above 3 was different for every mTC, the size of the overlap in genes between two mTCs changed. The significance of the Spearman correlation found between two mTCs, therefore, was dependent on the number of overlapping genes. Hence, the significance of the number of overlapping genes between mTCs should be determined. To this end, for a pair of mTCs, two sets of random gene identifiers were selected from all possible gene identifiers. The amount of randomly selected genes per set corresponded to the number of genes with a weight of > 3 in both mTCs. Subsequently, the overlap in gene identifiers between the two random sets of gene identifiers was determined. By repeating this 10,000 times, the chance of finding a given overlap between two sets of genes could be determined.

Ultimately, mTCs were said to be concordant when their correlation was > 0.5, with a *P*-value < 0.05, given that there was a significant overlap in genes (*P*-value of overlap < 0.05).

### Clustering of metabolic transcriptional components, genes, and samples

For each of the four datasets, the mixing matrix (MM) containing activity scores was clustered both on samples and mTCs. To this end, hierarchical clustering was performed using ward-D2 as the method and 1-cor(data) as the distance function. Heatmaps were created using R’s *gplots* package (v3.0.1). Based on the MM clustering for every dataset, metabolic subtypes were defined. To determine the sizes of clusters of samples that would make up a metabolic subtype, the dendrograms resulting from hierarchical clustering of the samples were systematically cut at dissimilarity values ranging from 0.0 to 8.0 with increments of 0.2. For each of the four datasets GEO, TCGA, CCLE, and GDSC, the cutoff was chosen at the dendrogram height at which the smallest cluster reached a size of 50 samples.

### CIBERSORT

Relative and absolute immune fractions for 22 immune cell types were estimated for all samples in GEO and TCGA datasets using the CIBERSORT algorithm running with default parameters, 1000 permutations, and selecting “absolute nosumto1” as output. This output was then associated with the activity of the mTCs using Spearman correlation.

### Statistical analyses

Univariate distant relapse-free survival (DRFS) analyses on breast cancer samples were performed using a Cox regression model through *survminer* (v0.4.3) and *survival* (v2.43-3) packages in R. Confidence intervals were set at 0.95. Significance was tested through the log rank test. Pearson correlations were performed in R using the cor.test()-function from the *stats* package (v.3.5.1). Spearman correlations and the corresponding exact *P*-values were calculated using the *pspearman* package (v0.3-0) in R, with a *t*-distribution as an approximation.

### Approximation of batch effects and tissue specificity of mTCs

First, the explained variance of every component from the perspective of a sample (as a percentage) was estimated using the squares of the mixing matrix weights of a sample divided by the sum of the squares. This explained variance matrix for samples was then summarized into a mean explained variance for studies by summarizing samples belonging to the same study (through the annotated GEO series accession number or TCGA tissue source site code). In the supplementary figures, only the highest explained variance available for any study is given. Similarly, tissue specificity was approximated by calculating the mean explained variance for tissue types by summarizing samples belonging to the same tissue subtype.

## Results

### A subset of transcriptional components is associated with metabolic processes

**Fig. 1:**
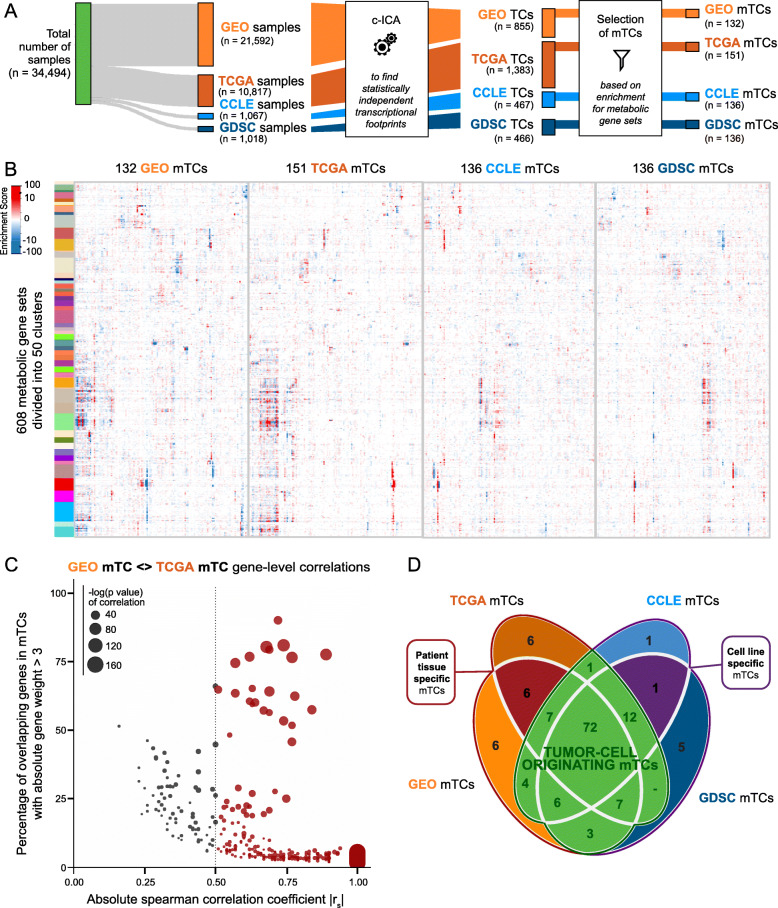
Identification of metabolic transcriptional components (mTCs). **A** Workflow for identification of mTCs. Consensus independent component analysis (c-ICA) is applied to identify transcriptional components (TCs). Subsequent systematic selection of TCs enriched for metabolic processes resulted in 132, 151, 136, and 136 mTCs for the GEO, TCGA, CCLE, and GDSC datasets, respectively. **B** Hierarchically clustered heatmaps showing the enrichment of the 608 metabolic gene sets of mTCs identified in the GEO, TCGA, CCLE, and GDSC datasets. **C** Scatter plot showing absolute Spearman correlation coefficients (*x*-axis) versus the percentage of overlapping top genes (genes with absolute weight > 3) between GEO mTCs and TCGA mTCs (*y*-axis). Only significant pair-wise correlations (with *P*-values < 0.05) are shown. Colored dots show the correlations > 0.5, the size of the dots represents the *P*-value of these Spearman correlations. The transparency of the dots is the same for all data points. Darker dot colors therefore mean that multiple data points are overlapping. **D** Venn diagram quantifying the overlap of mTCs between each dataset based on their pair-wise correlations. Two mTCs are counted as shared between datasets, when they have a high absolute Spearman correlation (|*r*_*s*_| > 0.5). Three groups of (shared) mTCs, mentioned in the text, are designated

Previously, we collected gene expression data from four databases: the Gene Expression Omnibus (GEO dataset, *n* = 21,592), The Cancer Genome Atlas (TCGA dataset, *n* = 10,817), the Cancer Cell Line Encyclopedia (CCLE dataset, *n* = 1067), and the Genomics of Drug Sensitivity in Cancer (GDSC dataset, *n* = 1018) (Fig. 1A), totaling 34,494 samples [17]. Overall, 28,200 expression profiles originated from patient-derived complex tissue cancer biopsies, 4209 from complex tissue biopsies of non-cancerous tissue, and 2085 from cell lines. The samples in these four databases encompass 89 cancer tissue types and subtypes and 19 non-cancerous tissue types. For GEO and CCLE datasets, the expression profiles were generated with Affymetrix HG-U133 Plus 2.0. Expression profiles within the GDSC dataset were generated with Affymetrix Human Genome U219, and TCGA profiles were generated with RNA sequencing.

Gene expression profiling measures the net expression level of individual genes, thus reflecting the integrated activity of underlying regulatory factors, including experimental, genetic, and non-genetic factors. To gain insight into the number and nature of these regulatory factors and their effects on gene expression levels, i.e., their transcriptional footprints, we previously performed consensus independent component analysis (c-ICA) on each of the abovementioned four datasets separately [17], resulting in four sets of transcriptional components (TCs). In every TC, each gene has a specific weight. This weight describes how strongly and in which direction the underlying transcriptional regulatory factor influences the expression level of that gene. c-ICA also provides a “mixing-matrix” per dataset, in which each column corresponds to a TC and each row corresponds to a sample. Values in the mixing matrix are interpreted as measurements of the activity of the TCs in an individual sample; we refer to these as “activity scores.” Ultimately, the analysis yielded 855, 1383, 467, and 466 TCs for GEO, TCGA, CCLE, and GDSC datasets, respectively (Fig. 1A).

Gene set enrichment analysis (GSEA) with 608 gene sets that describe metabolic processes was performed to identify TCs enriched for metabolic processes. The gene sets were selected from the gene set collections Biocarta (*n* = 7), the Kyoto Encyclopedia of Genes and Genomes (KEGG, *n* = 64), the Gene Ontology Consortium (GO, *n* = 508), and Reactome (*n* = 29) within the Molecular Signatures DataBase (MSigDB, v6.1; for the systematic selection strategy, see the “Methods” section). We performed consensus clustering on the enrichment scores of the 608 metabolic gene sets to identify potential biological redundancy in the metabolic gene set definitions (Supplementary Fig. 1). This resulted in 50 clusters of gene sets, which can be ascribed to different metabolic themes (Additional File 1). Based on these 50 enrichment clusters, 132 (GEO), 151 (TCGA), 136 (CCLE), and 136 (GDSC) mTCs were defined (Fig. 1A, B; see the “Methods” section for the systematic selection strategy). These mTCs represent the metabolic transcriptional footprints present in our broad set of samples, i.e., patient-derived samples, cancer cell line samples, and non-cancer samples. Every mTC is therefore enriched for at least one metabolic process, but it is also possible that multiple (possibly co-regulated) metabolic processes are represented by a single mTC. This is because the nature and effect of every regulatory factor that underlies a captured transcriptional footprint are different. Consequently, some mTCs might capture the transcriptional footprint of a metabolic process that is co-regulated with a non-metabolic process. Moreover, a metabolic process might even be represented by multiple mTCs, which are all differently active in specific tissue samples. The number of metabolic gene sets that are enriched per mTC is shown in Supplementary Fig. 2 and Additional File 1.

Some of the identified mTCs may capture the transcriptional footprints of experimental factors as well. Therefore, we investigated how much of the variance in activity scores of each mTC could be explained by experimental batches. For GEO mTCs, experimental batches were determined by the provided GSE identifiers (i.e., experiment series identifiers). For TCGA mTCs, experimental batches were determined by the tissue source site of samples (e.g., 2H, Erasmus MC, esophageal carcinoma). We observed that 12/132 GEO mTCs showed a potential putative batch effect with more than 10% explained variance (Supplementary Fig. 3A). However, six of the 12 GEO mTCs with a putative batch effect also explained more than 10% of the variance in the gene expression of samples belonging to a single tissue subtype (Supplementary Fig. 3A). One of the 151 TCGA mTCs showed a putative batch effect with 20.5% explained variance (Supplementary Fig. 3B). This mTC, TCGA mTC 43, also showed tissue specificity for thymoma, a tissue type that is not present in the GEO dataset. These observations might indicate that the mTCs showing a putative batch effect in fact describe tissue-specific biology of tissues that are only present in a single experiment in our dataset.

### Metabolic TCs are robust across different datasets and platforms

Pair-wise comparison of mTCs between datasets, based on gene weights, showed that 91–99% of mTCs per dataset were highly correlated (|*r*_*s*_| ≥ 0.5, *P*-value < 0.05 as a threshold) with at least one mTC identified in another dataset (Fig. 1C, D and Supplementary Fig. 4A-G). This indicates that most of the mTCs were cross-platform and cross-dataset robust.

Given the selected correlation threshold (|*r*_*s*_| ≥ 0.5, *P*-value < 0.05), 72 mTCs could be identified with a highly similar gene weight pattern in all four datasets (Fig. 1D). Thus, these mTCs capture a transcriptional footprint that is very similar in both patient-derived complex biopsies and cell lines. As cell lines lack a TME, these 72 mTCs were considered to capture metabolic processes that reflect tumor cell characteristics. Six GEO mTCs were identified that were highly correlated with TCGA mTCs, but not highly correlated with any CCLE or GDSC mTC (Fig. 1D). These mTCs, therefore, might capture transcriptional footprints that are specific for complex biopsies obtained from patient-derived cancer tissue and may originate from the TME or capture a transcriptional footprint from tissue only present in the GEO and TCGA datasets. One pair of mTCs was identified with a gene weight pattern that was highly similar in CCLE and GDSC datasets only, capturing a metabolic transcriptional footprint that could only be found in cell line models (Fig. 1D).

### Metabolic TCs identify new genes potentially involved in metabolic processes

**Fig. 2:**
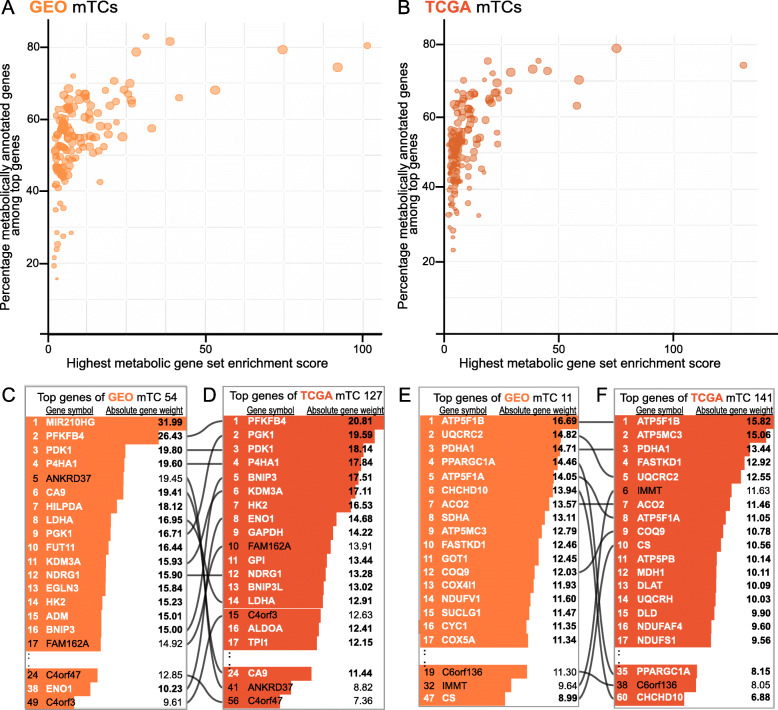
Metabolic TCs identify new genes potentially involved in metabolic processes. **A**, **B** Scatterplots showing the highest metabolic gene set enrichment score for every GEO (**A**) and TCGA (**B**) mTC (*x*-axis) versus the percentage of metabolically annotated genes among the top genes (genes with absolute weight > 3) in those mTCs. The size of the dots corresponds to the absolute amount of metabolically annotated genes in the corresponding mTC. The transparency of the dots is the same for all data points. Darker dot colors therefore mean that multiple data points are overlapping. **C**, **D** Top genes in GEO mTC 54 and TCGA mTC 127. Text colored white shows the genes that are a member of at least one of the 608 defined metabolic gene sets. Lines signify the genes that are the top genes in both GEO and TCGA mTCs. **E**, **F** Top genes in GEO mTC 11 and TCGA mTC 141

Among the “top” genes in every mTC—defined as the genes with an absolute weight of > 3 in an mTC—many genes were a member of the 608 metabolic gene sets (Fig. 2A, B, Supplementary Fig. 5). However, even for the mTCs with the absolute highest gene set enrichment scores for a metabolic gene set, at least 20% of top genes were not members of any of the metabolic gene sets. Because these genes were nevertheless part of an mTC, they may be potentially involved in the metabolic processes that showed enrichment.

For example, two strongly correlated mTCs, GEO mTC 54 and TCGA mTC 127 (|*r*_*s*_| = 0.77), both showed enrichment for glycolysis and the metabolic process of ADP (Fig. 2C, D, Additional File 1). GEO mTC 54 contained 262 top genes, of which 155 (59.1%) were also among the top genes in TCGA mTC 127. Both mTCs contained multiple top genes that are known targets of the HIF-1 complex and genes previously found to be part of a hypoxic signature [18]. Several top genes of both GEO mTC 54 and TCGA mTC 127 (e.g., *FAM162A*, *C4orf3*, *C4orf47*, and *ANKRD37*) are currently not a member of any of the 608 metabolic gene sets. However, these data suggest that these four genes are involved in glycolysis and are possibly hypoxia-related. Indeed, several studies have indicated that at least *FAM162A* and *ANKRD37* are regulated by the transcription factor HIF-1α [19, 20].

As a second example, we investigated two highly correlated mTCs, GEO mTC 11 and TCGA mTC 141 (*r*_*s*_ = 0.68), which showed enrichment for mitochondrial metabolic processes such as oxidative phosphorylation and the TCA cycle (Fig. 2E, F, Additional File 1). GEO mTC 11 contained 427 top genes, of which 270 (63.2%) were among the top genes in TCGA mTC 141. In these two mTCs, *C6orf136* and *IMMT* are examples of top genes currently not assigned to any of the 608 metabolic gene sets. *C6orf136* and *IMMT* were previously identified in functional mitochondria proteome profiles [21]. These results suggest that mTCs could assign metabolic functions to genes currently not members of known gene sets describing metabolic processes.

### Clustering sample activity scores of mTCs reveal multiple metabolic subtypes

**Fig. 3:**
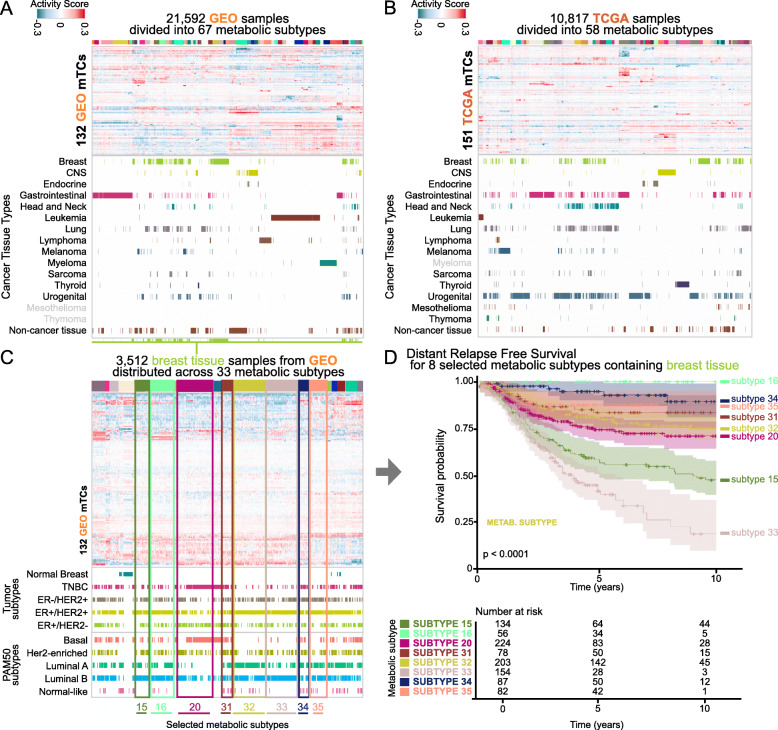
Clustering activity scores of mTCs reveal multiple metabolic subtypes. **A** A total of 21,592 GEO samples were hierarchically clustered based on the activity scores of mTCs and divided into 67 metabolic subtypes. **B** A total of 10,817 TCGA samples were hierarchically clustered based on the mTC activity scores and divided into 58 metabolic subtypes. **C** Metabolic landscape of the subset of breast tissue samples in the GEO dataset. Subtypes with DFS data that were selected for survival analysis are highlighted. Gray labels designate the tissue types that are present in other datasets but are not present in the given dataset. **D** Distant relapse-free survival of breast cancer patients in the GEO dataset. Patient-derived samples were stratified per metabolic subtype. Kaplan-Meier curves are shown with a confidence interval of 0.95

To investigate the heterogeneity of the metabolic transcriptome in a broad range of cancer subtypes, we hierarchically clustered the mixing matrix provided by consensus-ICA that contains the activity score of mTCs in every sample (Fig. 3A, B and Supplementary Fig. 6A, B). We selected the cutoff heights of the resulting dendrograms so that every cluster—referred to as metabolic subtype—contained at least 50 samples (Supplementary Fig. 6C and D). This clustering analysis divided the 21,592 GEO samples into 67 metabolic subtypes with a median of 276 samples per subtype (range 54–1252) and the 10,817 TCGA samples into 58 metabolic subtypes with a median of 167 samples per subtype (range 52–536). For an overview of the metabolic subtypes and their sample composition, see Supplementary Fig. 7, 8, and Additional File 2. Two types of patterns emerged.

The first pattern consisted of tumor types with samples that belong to one dominant metabolic subtype. For example, 102/133 (76.7%) of thyroid cancer samples in the GEO dataset fell into one metabolic subtype (subtype 27, Supplementary Fig. 7, Additional File 2). Similarly, 446/509 (87.6%) of thyroid cancer samples in the TCGA dataset fell into metabolic subtype 43 (Supplementary Fig. 8, Additional File 2). In line with the biology of thyroid tissue, both GEO metabolic subtype 27 and TCGA metabolic subtype 43 were characterized by high activity scores of mTCs enriched for thyroid hormone metabolism (GEO mTC 64 and TCGA mTC 87; Additional File 1).

The second pattern consisted of several tumor types that were not characterized by a few dominant metabolic subtypes. Instead, their samples were divided across multiple metabolic subtypes. For example, the 3512 breast cancer samples in the GEO dataset were divided across 33 metabolic subtypes (Fig. 3C). These metabolic subtypes did not follow the breast cancer classification based on ER and HER2 receptor status, nor did they align with the classification based on PAM50 molecular subtypes (Fig. 3C, Additional Files 2 and 3). In line with this observation in the GEO dataset, the 1100 breast cancer samples in the TCGA dataset were also scattered across 29 metabolic subtypes.

Several metabolic subtypes likewise contained samples from multiple tumor types. For example, GEO metabolic subtype 22 contained samples from 25 tumor types, including 42 ovarian cancer samples (22% of all ovarian cancer), 33 synovial sarcoma samples (97% of all synovial sarcoma), and 15 Ewing’s sarcoma samples (58% of all Ewing’s sarcoma; Supplementary Fig. 7 and Additional File 2). GEO mTC 111 had the highest absolute median activity score in GEO metabolic subtype 22 (Additional File 2). This mTC showed enrichment for the metabolism of nicotinamide adenine dinucleotide phosphate (NADP) and genes involved in the activation of an innate immune response (Additional File 1).

These results show that the classification of samples based on metabolic subtype yields different patterns than current classification systems based on receptor status or PAM50 subtypes in breast cancer.

### Metabolic subtypes are associated with distant relapse-free survival in breast cancer

We then investigated if metabolic subtypes could have clinical relevance. We had previously collected distant relapse-free survival (DRFS) data for 1207 breast cancer samples [22]. As mentioned earlier, breast cancer samples in the GEO dataset were divided across 33 of the 67 metabolic subtypes. Of these 33 subtypes, eight contained > 50 breast cancer samples with data available for DRFS: subtypes 15, 16, 20, 31, 32, 33, 34, and 35. We found that patients from breast cancer samples assigned to metabolic subtypes 16 and 33 showed the best and worst DRFS, respectively (*P*-value = 1.08 × 10^−23^, log-rank test; Fig. 3D). Distributions of the standard prognostic factors within these eight metabolic subtypes are presented in Additional File 3. Metabolic subtypes might be correlated with standard factors such as age, p53 status, and lymph node involvement, which can explain the survival differences. Although the metabolic subtypes might not be statistically independent prognostic factors in breast cancer, their association with DRFS could be helpful to understand the biology that is potentially driving breast cancer behavior.

### The activity of mTCs is associated with drug sensitivity

The CCLE and GDSC databases contain the sensitivities of cell lines to a large panel of drugs expressed as IC_50_ values. With a threshold of |*r*_*s*_| > 0.2, we found associations between the activity scores of 61 CCLE mTCs, 90 GDSC mTCs, and the IC_50_ values of 238 drugs (Additional File 4).

**Fig. 4:**
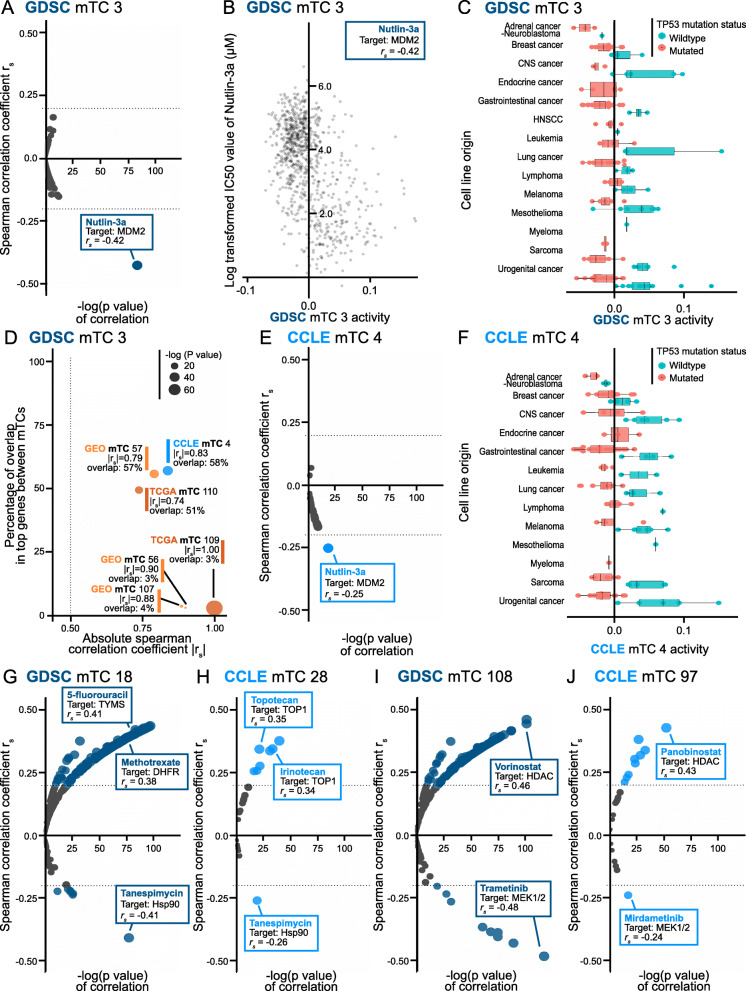
Associations between mTCs and drug sensitivity for the selected examples. **A** Spearman correlations between drug IC_50_ values and the activity of GDSC mTC 3. **B** Scatter plot showing the association between the (log-transformed) IC_50_ value of nutlin-3a and the activity of GDSC mTC 3 in samples. The transparency of the dots is the same for all data points. Darker dot colors therefore mean that multiple data points are overlapping. **C** Box plot of the activity of GDSC mTC 3 across cell lines, colored for their *TP53* mutation status. **D** Pair-wise correlations between GDSC mTC 3 and mTCs from the GEO, TCGA, and CCLE datasets. Every dot corresponds to an mTC with a correlation to GDSC mTC 3 of ≥ 0.5. Dot sizes correspond to the *P*-value of the Spearman correlation coefficient; the *y*-axis gives the percentage of the overlapping top genes between the two mTCs involved in the correlation. **E** Spearman correlations between the drug IC_50_ values and the activity of CCLE mTC 4. **F** Box plot of the activity of CCLE mTC 4 across cell lines, colored for their *TP53* mutation status. **G**–**J** Spearman correlations between the drug IC_50_ values and the activity of GDSC mTC 18, CCLE mTC 28, GDSC mTC 108, and CCLE mTC 97, respectively

For example, in the GDSC dataset, an increase in activity score of GDSC mTC 3 was associated with a decrease in IC_50_ value of (i.e., increased sensitivity to) nutlin-3a (|*r*_*s*_| = 0.42; Fig. 4A, B). Nutlin-3a targets the p53 pathway through inhibition of MDM2. In line with this, GDSC mTC 3 showed strong enrichment for genes involved in the p53 pathway, with *MDM2* ranked as the second gene (Additional File 1). GDSC mTC 3 was strongly correlated with CCLE mTC 4 (|*r*_*s*_| = 0.84), GEO mTC 57 (|*r*_*s*_| = 0.79), and TCGA mTC 110 (|*r*_*s*_| = 0.74) (Fig. 4D), suggesting that this mTC was captured in cell line datasets as well as in the two patient-derived datasets. Indeed, an increase in activity score of CCLE mTC 4 was associated with a decrease in IC_50_ value of nutlin-3a as well (|*r*_*s*_| = 0.25; Fig. 4E). Cell lines with wild-type *TP53* had a higher activity score of GDSC mTC 3 (Fig. 4C). Also, cell lines with wild-type *TP53* had a higher activity score of CCLE mTC 4 (Fig. 4F).

In another example, the activity score of GDSC mTC 18 was found to be associated with the IC_50_ values of 142 drugs (|*r*_*s*_| range 0.20–0.44; Fig. 4G). An increase in the activity score of GDSC mTC 18 in a sample was associated with a higher IC_50_ value (i.e., increased resistance) for 135 of these drugs, including the widely used DNA synthesis-inhibiting antimetabolites 5-fluorouracil (|*r*_*s*_| = 0.41) and methotrexate (|*r*_*s*_| = 0.38). GDSC mTC 18 was strongly correlated with CCLE mTC 28 (|*r*_*s*_| = 0.84), GEO mTC 35 (|*r*_*s*_| = 0.59), and TCGA mTC 58 (|*r*_*s*_| = 0.55), indicating that this mTC is also captured in both cell line datasets and the two patient-derived datasets. CCLE mTC 28 was associated with a higher IC_50_ value (i.e., increased resistance) for 7 drugs including topoisomerase inhibitors topotecan (|*r*_*s*_| = 0.35) and irinotecan (|*r*_*s*_| = 0.34) (Fig. 4H). In line with this, GDSC mTC 18 was associated with increased resistance to SN38, the active metabolite of irinotecan (|*r*_*s*_| = 0.21). All four of the highly correlated mTCs were enriched for genes involved in glutathione metabolism, the metabolism of cellular ketones and xenobiotics, and drug detoxification (Additional File 1). Specifically, genes belonging to the aldo-keto reductase family 1 (*AKR1*) were among the top genes in these mTCs. Previous studies have reported a role for the glutathione system in resistance to irinotecan and 5-fluorouracil [23], and specifically, a role for the *AKR1* family in resistance to, e.g., methotrexate and irinotecan [24, 25]. In contrast, we observed that an increased activity score of GDSC mTC 18 was associated with a decrease in IC_50_ value (i.e., increased sensitivity) for only seven drugs (|*r*_*s*_| range 0.20–0.41; Fig. 4G). The drug with the highest negative correlation was tanespimycin (17-AAG), an Hsp90 inhibitor (|*r*_*s*_| = 0.41). An increased activity score of CCLE mTC 28 was associated with a decrease in IC_50_ value for tanespimycin as well (|*r*_*s*_| = 0.26; Fig. 4H). A direct link between the functions of glutathione and Hsp90 in oxidative stress has been suggested, as well as a relationship between tanespimycin sensitivity and *NQO1* expression, a gene coding for an enzyme reducing quinones to hydroquinones that is involved in detoxification [26, 27]. In line with these findings, we found that the *NQO1* gene is present near the top of GDSC mTC 18, CCLE mTC 28, GEO mTC 35, and TCGA mTC 58.

In a third example, increased activity of GDSC mTC 108 was associated with a lower IC_50_ value (i.e., increased sensitivity) to the MEK inhibitor trametinib (|*r*_*s*_| = 0.48) and a higher IC_50_ value (i.e., increased resistance) to the histone deacetylase inhibitor vorinostat (|*r*_*s*_| = 0.46; Fig. 4I and Additional File 4). GDSC mTC 108 was correlated with CCLE mTC 97 (|*r*_*s*_| = 0.32). Consistent with the observation for GDSC mTC 108, we found that increased activity of CCLE mTC 97 was associated with a lower IC_50_ value (i.e., increased sensitivity) to the MEK inhibitor mirdametinib (|*r*_*s*_| = 0.24) and a higher IC_50_ value (i.e., increased resistance) to the histone deacetylase inhibitor panobinostat (|*r*_*s*_| = 0.43; Fig. 4J and Additional File 4). This contrasting sensitivity for MEK and histone deacetylase inhibition is in line with the data from a study that used *BRAF*-mutated melanoma cell lines. The authors showed that cell lines with acquired resistance to MEK inhibitors subsequently became sensitive to treatment with the histone deacetylase inhibitor vorinostat [28]. They concluded that the MEK inhibitor resistance mechanism results from the activation (or reactivation) of MAPK cascades [29]. These findings are in line with our observation that both GDSC mTC 108 and CCLE mTC 97 were enriched for genes involved in the negative regulation of the MAPK cascade (Additional File 1).

As a final example, GDSC mTC 13 was enriched for genes involved in glutathione metabolism (e.g., *GSTO1*, *GSTP1*, and *ESD* were among the top-ranked genes in GDSC mTC 13). This mTC showed specifically high activity scores in primary effusion lymphoma cell lines BC-1, JSC-1, and CRO-AP2 (Supplementary Fig. 9A). An increase in the activity of this mTC in those cell lines corresponded to a decrease in the expression of genes involved in glutathione metabolism. Indeed, in a previous study, glutathione *S*-transferases were found to be specifically downregulated in patient-derived primary effusion lymphoma cells [30]. The activity of GDSC mTC 13 showed a negative correlation with the IC_50_ values of 117 drugs, among which metabolically targeted drugs methotrexate (*r*_*s*_ = − 0.37) and phenformin (*r*_*s*_ = − 0.29; Supplementary Fig. 9B and Additional File 4). This means that cell lines with a high activity of GDSC mTC 13 showed a low IC_50_ for these 117 drugs, i.e., were sensitive to them.

These examples demonstrate how mTCs can capture cross-dataset robust metabolic transcriptional footprints relevant for drug response.

### The activity of mTCs is associated with the immune composition of the tumor microenvironment

We determined the association between the activity of mTCs and the immune composition of the TME (Additional File 5; see the “Methods” section for details). The immune composition for all samples in the GEO and TCGA datasets was determined by inferring fractions of 22 immune cell types using the CIBERSORT algorithm [31]. We observed that the mTCs that were correlated with immune cell fractions could be divided into two groups. The first group included mTCs that were only identified in the patient-derived datasets. The second group contained mTCs that were identified in both the patient-derived and the cell line datasets.

**Fig. 5:**
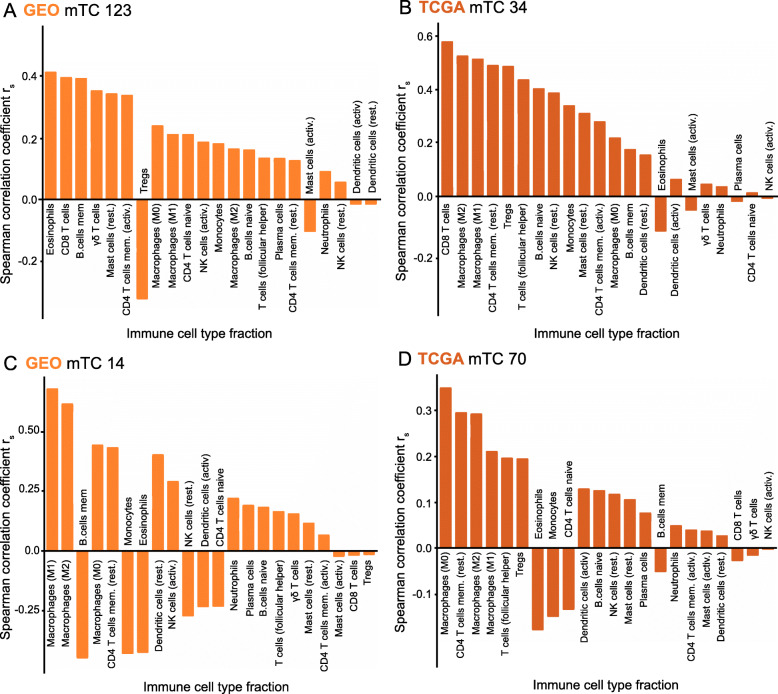
Associations between mTCs and the composition of the immune tumor microenvironment for selected examples. **A**, **B** Spearman correlations between the CIBERSORT-estimated immune cell fractions and the activity of GEO mTC 123 and TCGA mTC 34. **C**, **D** Spearman correlations between the CIBERSORT-estimated immune cell fractions and the activity of GEO mTC 14 and TCGA mTC 70

For example, the activity score of GEO mTC 123 was associated with estimated fractions of CD8+ T cells (|*r*_*s*_| = 0.40), γδ T cells (|*r*_*s*_| = 0.36), activated CD4 memory T cells (|*r*_*s*_| = 0.34), and regulatory T cells (|*r*_*s*_| = 0.32, Fig. 5A). Belonging to the group of mTCs only identified in the patient-derived datasets, GEO mTC 123 was correlated highly with only TCGA mTC 34 (|*r*_*s*_| = 0.28). In line with this, the activity score of TCGA mTC 34 was also associated with CD8+ T cell fractions (|*r*_*s*_| = 0.58, Fig. 5B). Both GEO mTC 123 and TCGA mTC 34 showed enrichment for genes involved in immunological processes such as leukocyte activation and cytokine metabolism and metabolic processes such as phosphatidylinositol and phospholipid metabolism (Additional File 1). Both GEO mTC 123 and TCGA mTC 34 have no high correlation with the mTCs in the cell line datasets, suggesting that these mTCs capture transcriptional activity that is not reflected in cell line cultures. Another possibility is that these mTCs capture transcriptional activity from non-cancerous (immune) cells in the TME.

GEO mTC 14 is illustrative of the second group of mTCs correlated with immune cell fractions and identified in both the patient-derived and the cell line datasets. The activity scores of GEO mTC 14 were correlated with the fractions of M1 macrophages (|*r*_*s*_| = 0.65) and M2 macrophages (|*r*_*s*_| = 0.59; Fig. 5C). GEO mTC 14 was correlated with TCGA mTC 70 (|*r*_*s*_| = 0.44), CCLE mTC 124 (|*r*_*s*_| = 0.47), and GDSC mTC 33 (|*r*_*s*_| = 0.33). All four mTCs were enriched for genes involved in the metabolism of extracellular macromolecules (Additional File 1). Genes coding for several types of collagens were among the top-ranked in these mTCs. This is in line with previous reports indicating that macrophages can function as collagen-producing cells in the TME [32]. GEO mTC 14 and TCGA mTC 70 showed a high activity score in subsets of breast cancers, lung cancers, and sarcomas (Supplementary Fig. 10A, B). A negative activity score of GEO mTC 14 and TCGA mTC 70 was observed in a subset of hematological cancers and hematological cancer cell lines in both GDSC and CCLE mTCs. These mTCs were present in both patient datasets and cell line datasets, indicating that the captured metabolic processes reflect tumor cell characteristics active under cell culture conditions and that these processes are associated with the fraction of macrophages present in the immune TME of patient-derived samples.

Genes involved in the metabolism of immune cells are also among the top-ranked genes in mTCs that are correlated with immune cell fractions. For example, *ACOD1*, aconitate decarboxylase 1, had the fourth highest weight in GEO mTC 56, which was enriched for genes involved in the inflammatory response. Other high-ranking genes in GEO mTC 56 were for example *TNF* (#1), *CCL4* (#2), and several other genes known to be involved in immune signaling. Next to this immunological process, GEO mTC 56 was enriched for the metabolic process of reactive oxygen species metabolism regulation (Additional File 1). GEO mTC 56 was correlated with CCLE mTC 134 (|*r*_*s*_| = 0.46) and GDSC mTC 29 (|*r*_*s*_| = 0.40). Moreover, the activity of GEO mTC 56 was highly correlated with the determined eosinophil fraction in a sample (|*r*_*s*_| = 0.43; Supplementary Fig. 11). The production of reactive oxygen species is a known effector function of eosinophils [33].

These examples show that by correlating inferred immune cell fractions of samples with the activity scores of mTCs in samples, the relationship between the metabolic transcriptome and the various components of the immune TME could be assessed.

## Discussion

We used the wealth of publicly available pan-cancer transcriptomic data to study human metabolism on a large scale. Previous work used either single-cell sequencing or bulk cell transcriptomic profiles to study the metabolism of specific cancer types [34, 35], or pan-cancer, but based on a single platform [36, 37]. Our present study differs from this previous work in two essential aspects. Firstly, we used c-ICA to segregate the average expression patterns of complex biopsies into statistically independent components [38]. Previous studies investigated the average gene expression profiles of complex biopsies and can therefore only distinguish the gene expression signature and regulation of more pronounced metabolic processes. With c-ICA, it is possible to identify statistically independent regulatory factors and their transcriptional footprints and distinguish pronounced from more subtle metabolic processes. In this way, this analysis demonstrates the complex nature of metabolically relevant transcriptional footprints and their heterogenous activity in samples and cell lines. It enabled us to determine the association of both pronounced and subtle metabolic processes with, e.g., patient outcome and the composition of the TME in a complex biopsy. Secondly, the present study is the most extensive transcriptional analysis of metabolism and the first that integrated patient-derived data from GEO and TCGA with cell line data from CCLE and GDSC. The samples in these four datasets were obtained from a multitude of independently constructed, publicly available cohorts, and the expression profiles were generated using different technologies (microarray or RNA-sequencing). This integrated dataset enabled us to demonstrate that most of the identified mTCs were robust and independent from dataset-specific and platform-specific characteristics. The observed overlap, or lack of overlap, between patient-derived and cell line-derived mTCs can help researchers understand how metabolic genes and pathways identified in cell lines can be translated to a patient tissue context and vice versa.

Furthermore, we hypothesize that metabolic processes identified only in patient-derived samples and not in cell line samples capture metabolic processes that are in part driven by growth conditions specific to the TME, which are not reflected in cell cultures. Potentially, these metabolic processes could originate from cells in the TME. These microenvironment-specific metabolic processes will not be captured by mTCs in cell line datasets. This is because bulk expression profiles of cancer cell line samples do not harbor transcriptional footprints associated with non-cancerous cells.

The metabolic landscape enabled us to classify samples based on the transcriptional activity of metabolic processes, resulting in metabolic subtypes. However, this metabolic classification was often not in full alignment with current classification systems based on aspects such as receptor status and PAM50 molecular subtyping. We demonstrated that metabolic subtypes were associated with disease outcomes for breast cancer, emphasizing the relevance of metabolic pathway-based classification in cancer. The heterogeneity (metabolic and otherwise) within and between cancer types is well recognized, and alternative subtyping based on metabolite profiling and the metabolic transcriptome have been proposed before [37, 39]. More specifically, clinically significant metabolism-based classifications have been proposed in breast cancer [40, 41]. The most active mTCs in a metabolic subtype relevant to disease outcome could thus be used to generate new hypotheses for treatment targets. Additionally, the association between the activity of mTCs and drug sensitivity could help to design these future therapeutic strategies.

Metabolic heterogeneity and plasticity are not limited to cancer cells but are also applicable to the immune cells present in the tumor microenvironment. Immune cells undergo metabolic changes when activated, and their metabolic status can overlap with the metabolic state of cancer cells [42]. For example, the Warburg effect is classically seen as an example of a metabolic transformation in cancer cells. However, it is also observed in activated T cells [43, 44]. In the context of metabolism, this complex interplay between cancer cells and immune cells present in the microenvironment gives a new dimension to the use of drugs that target metabolic processes [45, 46]. For instance, modulating metabolism in T cells from glycolytic to an OXPHOS-weighted profile has been shown to improve immunotherapy [47, 48]. Our transcriptional metabolic landscape can contribute to knowledge of immunometabolism and, combined with the association of mTCs with drug sensitivity, can also contribute to the formulation of new hypotheses on how to metabolically engage the tumor and its immune microenvironment, thus improving the response to immunotherapy.

Further research to gain an even more comprehensive understanding of the metabolism in patient-derived cancer samples should ideally integrate genomics, transcriptomics, proteomics, and metabolomics to capture the complexity of metabolic processes within cancer cells [49]. Recent initiatives are the Recon3D, Edinburgh Human Metabolic Network (EHMN), and Human1 projects [50–52]. However, challenges for these initiatives lie in the limited set of samples for which these high-dimensional multi-omics features are available and the use of predominantly cell line samples. Paired datasets on a large scale are needed to unleash the full potential of such an integrated approach.

To facilitate the use of our transcriptional metabolic landscape, we have provided access to all data via a web portal (www.themetaboliclandscapeofcancer.com). In this portal, users can explore genes, metabolic processes, and tissue types of interest. We invite researchers and clinicians to use this portal as a guide to the metabolic transcriptome in cancer or as a starting point for further research into cancer metabolism. In this manuscript, we have presented observations that align with experimental findings which were already published, demonstrating the validity of our approach. We look eagerly forward to upcoming experimental validations of the novel associations that could be put forward by investigating mTCs as well. These validations will further affirm the use of mTCs in understanding the complex associations of the metabolic transcriptome with, e.g., drug sensitivities and ultimately patient outcome.

## Conclusions

In the present study, we used consensus independent component analysis (c-ICA) in combination with gene set enrichment analysis (GSEA) to identify a broad set of robust metabolic transcriptional components (mTCs). The transcriptional metabolic landscape of patient-derived cancer tissue, cancer cell lines, and non-cancer samples was captured in these mTCs. We also showed how mTCs could be used to generate hypotheses by exploring associations between metabolic processes and drug sensitivities, patient outcomes, and the composition of the immune tumor microenvironment.

## Supplementary Information

**Additional file 1.A** 608 selected metabolic gene sets, divided into 50 gene set clusters. **B** Gene set enrichment scores of metabolic gene sets, for GEO mTCs -- Both for mTCs and gene sets, the amount of absolute enrichment scores > 3 are quantified. **C** Gene set enrichment scores of metabolic gene sets, for TCGA mTCs -- Both for mTCs and gene sets, the amount of absolute enrichment scores > 3 are quantified. **D** Gene set enrichment scores of metabolic gene sets, for CCLE mTCs -- Both for mTCs and gene sets, the amount of absolute enrichment scores > 3 are quantified. **E** Gene set enrichment scores of metabolic gene sets, for GDSC mTCs -- Both for mTCs and gene sets, the amount of absolute enrichment scores > 3 are quantified.

**Additional file 2.A** Tissue type composition of metabolic subtypes as defined for the GEO dataset. **B** Tissue type composition of metabolic subtypes as defined for the TCGA dataset. **C** Median activity score of GEO mTCs in GEO metabolic subtypes. **D** Median activity score of TCGA mTCs in TCGA metabolic subtypes.

**Additional file 3.A** Clinicopathological parameters of breast cancer tissue samples with DRFS survival data in the GEO dataset, stratified per metabolic subtype. Given are only those clusters with >50 breast cancer samples. **B** Distribution of PAM50 molecular intrinsic subtype of breast cancer tissue samples, stratified per metabolic subtype. Given are only those clusters with >50 breast cancer samples.

**Additional file 4.A** Spearman correlation coefficients between the IC_50_ values of drugs and the activity of CCLE mTCs in cell lines. **B** Spearman correlation coefficients between the IC_50_ values of drugs and the activity of GDSC mTCs in cell lines. **C** Spearman correlation coefficients between the IC_50_ values of metabolically targeted drugs and the activity of GDSC mTCs in cell lines.

**Additional file 5.A** Spearman correlation coefficients between the CIBERSORT estimated immune fractions and the activity of GEO mTCs in a sample. **B** Spearman correlation coefficients between the CIBERSORT estimated immune fractions and the activity of TCGA mTCs in a sample.

**Additional file 6.** Supplementary notes, figures, and methods.

## Data Availability

The data supporting the conclusions of this article is available at http://themetaboliclandscapeofcancer.com. The code is available at github.com/MetabolicLandscape Further information and requests for resources should be directed to and will be fulfilled by the lead contact, Rudolf S.N. Fehrmann (r.s.n.fehrmann@umcg.nl).
