# Supplementary material for: Robust metabolic transcriptional components in 34,494 patient-derived cancer-related samples and cell lines
Source: Cancer Metab. 2021 Sep 26;9:35. doi: 10.1186/s40170-021-00272-7 (PMC8474886; doi:10.1186/s40170-021-00272-7)
Supplement: Supplementary file 6 — Additional file 6. Supplementary notes, figures, and methods. [file 40170_2021_272_MOESM6_ESM.docx]

**Robust metabolic transcriptional components in 34,494 patient-derived cancer-related samples and cell lines**

V.C. Leeuwenburgh^1,2,#^, C.G. Urzúa-Traslaviña^1,#^, A. Bhattacharya^1^, M.T.C. Walvoort^2^, M. Jalving^1^, S. de Jong^1^, R.S.N. Fehrmann^1^

^1^ Department of Medical Oncology, Cancer Research Center Groningen, University Medical Center Groningen, University of Groningen, Groningen, the Netherlands

^2^ Department of Chemical Biology, Stratingh Institute for Chemistry, University of Groningen, the Netherlands

^#^ Contributed equally

**Supplementary Note**

Contains:

- Supplementary Figures 1 – 9 and their legends

- Supplementary Methods

- Additional Files 1 – 5 (large tables) are available as excel files.

**Supplementary Figures**

**
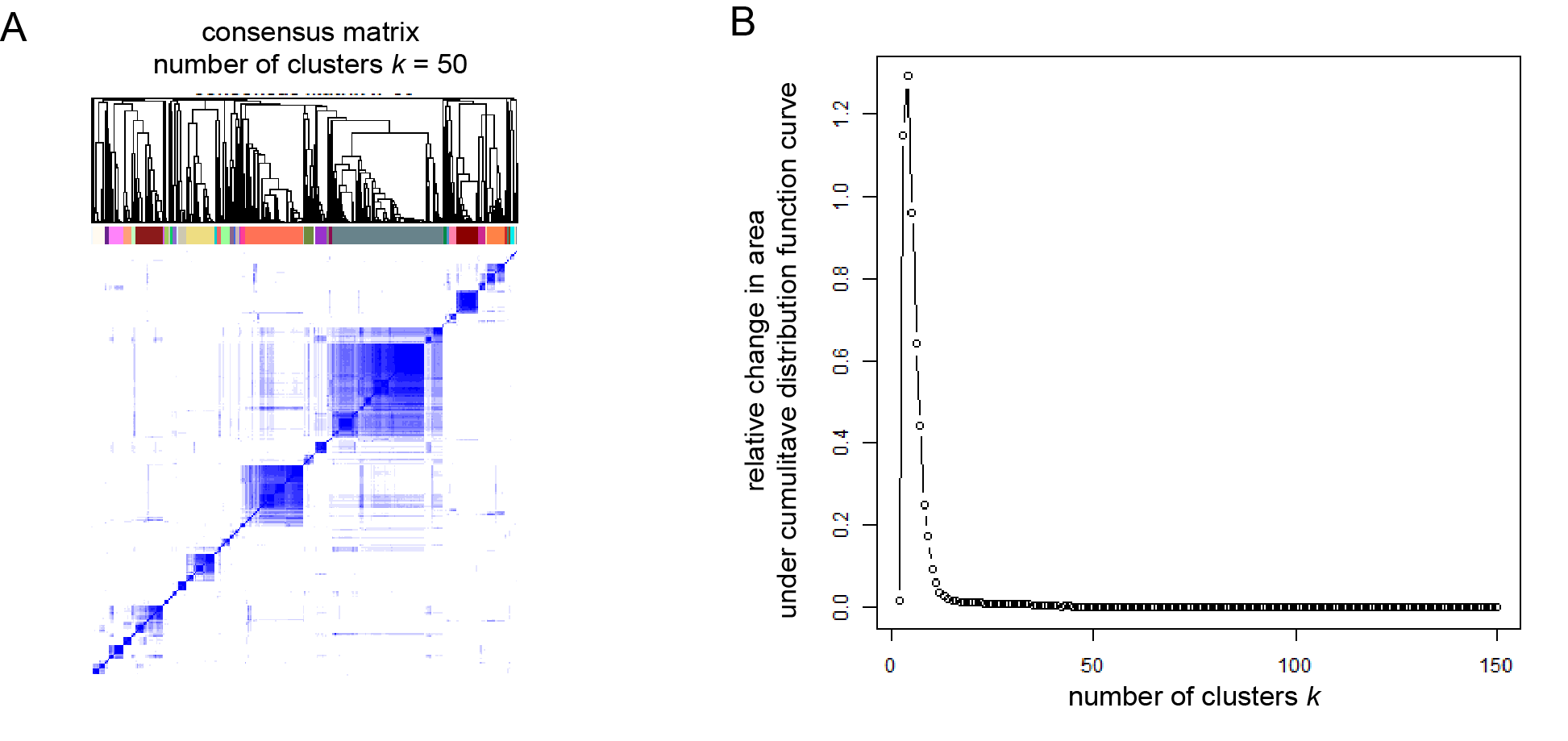
**

**Supplementary Fig. 1**

**(A)** Consensus clustering gene set enrichment scores of all TCs in the GEO dataset. Consensus matrix for a *k* of 50 gene set clusters. **(B)** Consensus clustering gene set enrichment scores of all TCs in the GEO dataset. Relative change in area under the consensus cumulative distribution function (CDF) curve with increasing *k*.

***
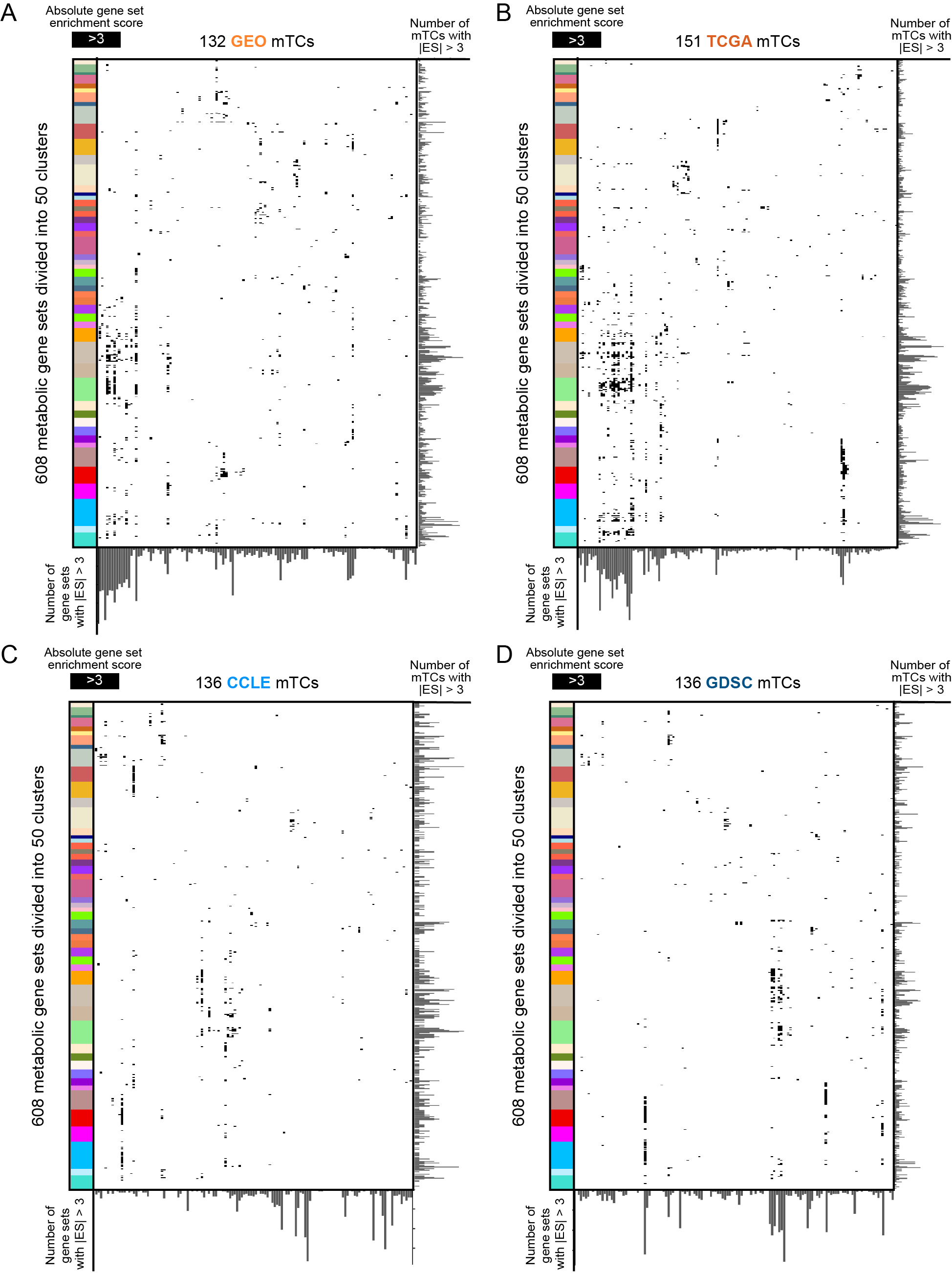
***

**Supplementary Fig. 2**

High-contrast heatmaps showing the metabolic gene sets with an absolute enrichment score |ES| > 3 (colored black) in all GEO **(A)**, TCGA **(B)**, CCLE **(C)** and GDSC **(D)** mTCs. The bar graphs on the x-axes show for every mTC how many of the 608 metabolic gene sets have an absolute enrichment score |ES| ≥ 3. The bar graphs on the y-axes show how many of the metabolic mTCs have an absolute enrichment score |ES| ≥ 3 for every gene set.

***
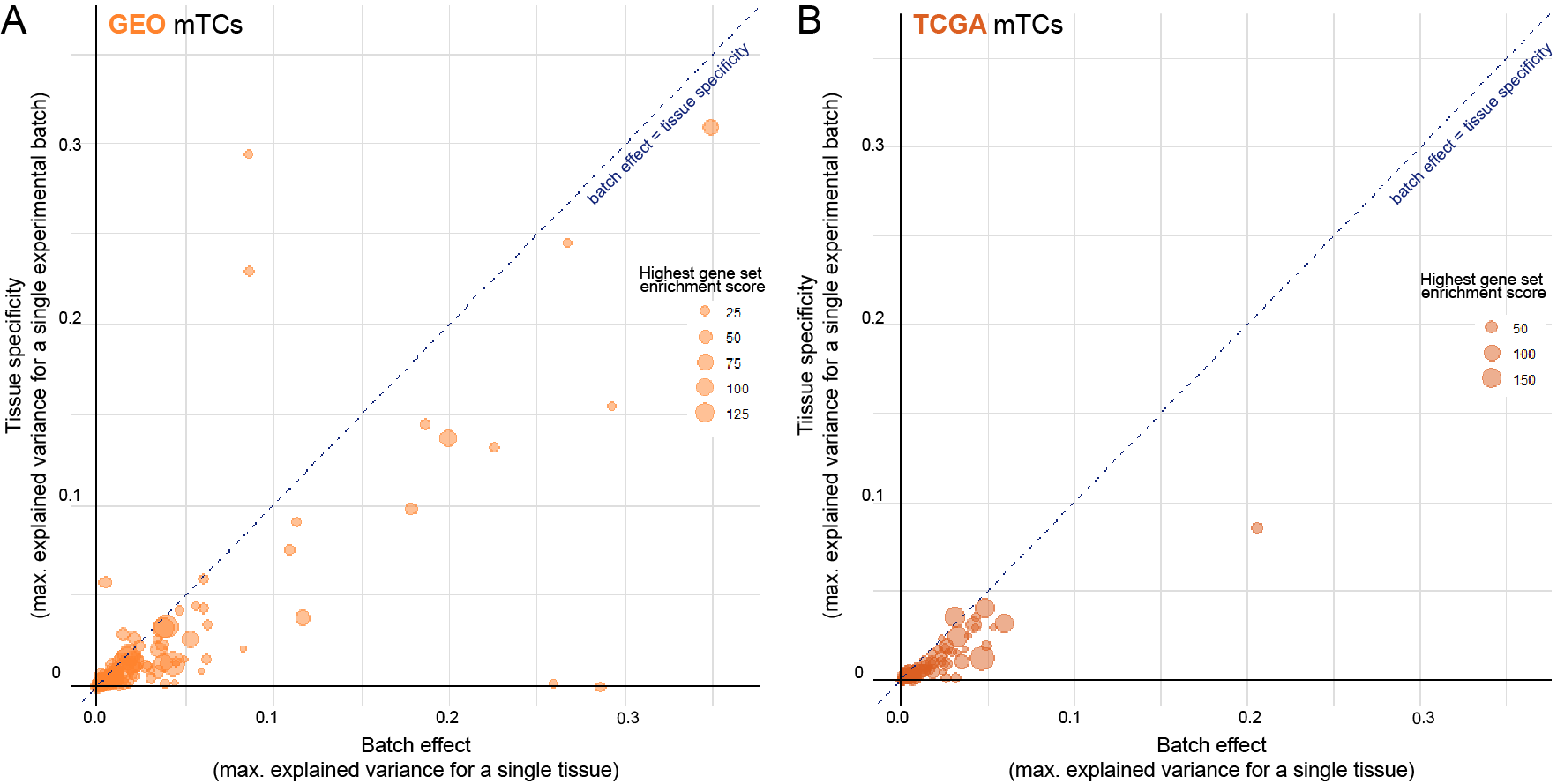
***

**Supplementary Fig. 3**

Scatter plots showing the maximum batch effect and tissue specificity for GEO **(A)** and TCGA **(B)** mTCs. Size of the dots correspond to the highest gene set enrichment score of that mTC. The transparency of the dots is the same for all data points. Darker dot colors therefore mean that multiple data points are overlapping. The magnitude of the batch effect in an mTC is estimated by the maximum fraction of the sample variance in an experimental batch that is explained by that mTC. Similarly, the tissue specificity of an mTC is estimated by the maximum fraction of the sample variance in a tissue type that is explained by that mTC.

***
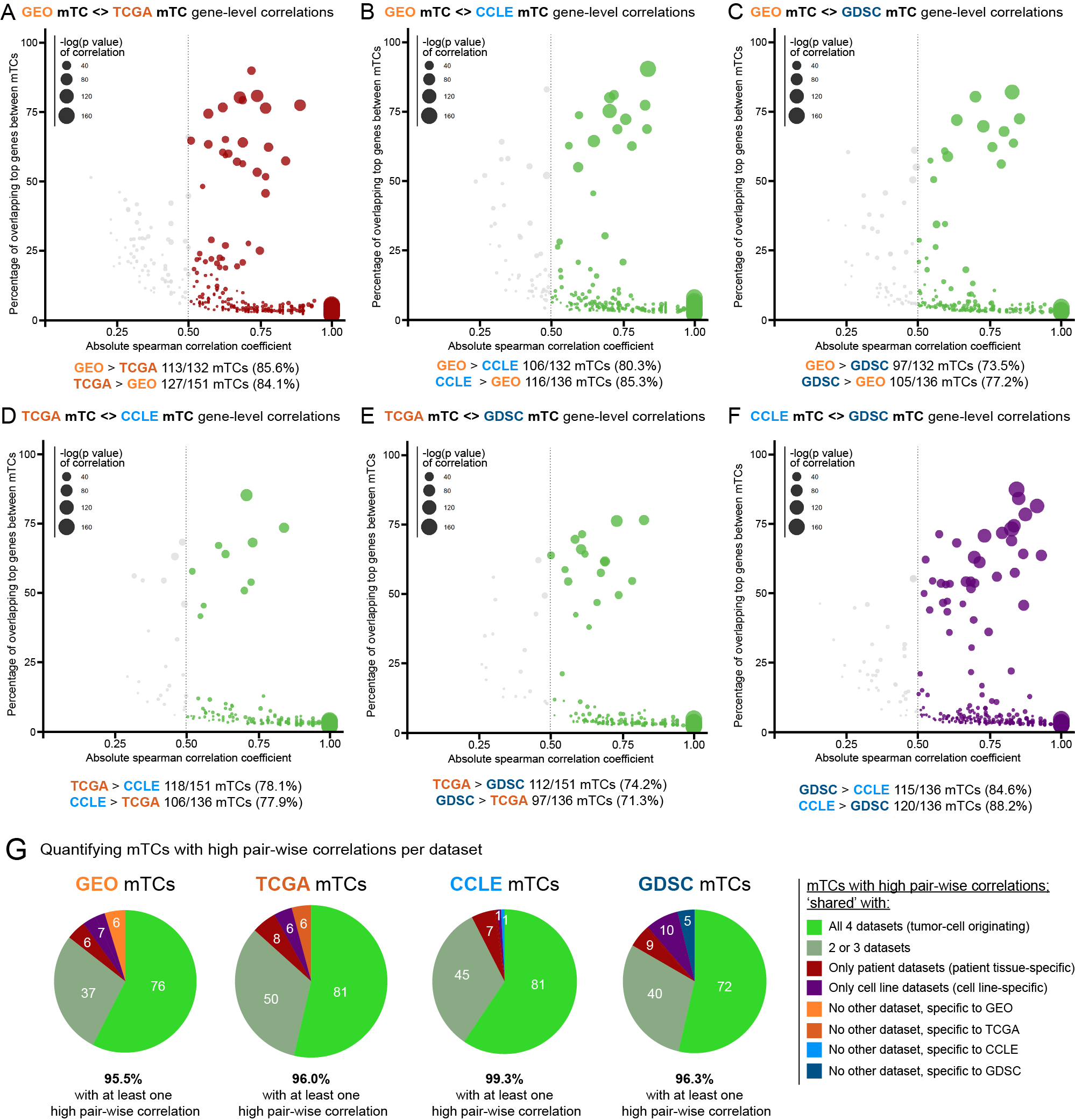
***

**Supplementary Fig. 4**

Scatter plot showing absolute spearman correlation coefficients (x-axis), versus the percentage of overlapping top genes (genes with absolute weight >3) between mTCs from different datasets (y-axis). Only significant pair-wise correlations (with P-value <0.05 and top gene overlap significance <0.05) are shown. Colored dots show absolute correlations > 0.5, the size of the dots represent the P-value of these spearman correlations. The transparency of the dots is the same for all data points. Darker dot colors therefore mean that multiple data points are overlapping. Scatter plots are shown for correlations between **(A)** GEO and TCGA mTCs, **(B)** GDSC and CCLE mTCs, **(C)** GEO and GDSC mTCs, **(D)** TCGA and GDSC mTCs, **(E)** GEO and CCLE mTCs, **(F)** TCGA and CCLE mTCs. **(G)** Pie graphs quantifying the amount of mTCs with high correlations for every dataset.

***
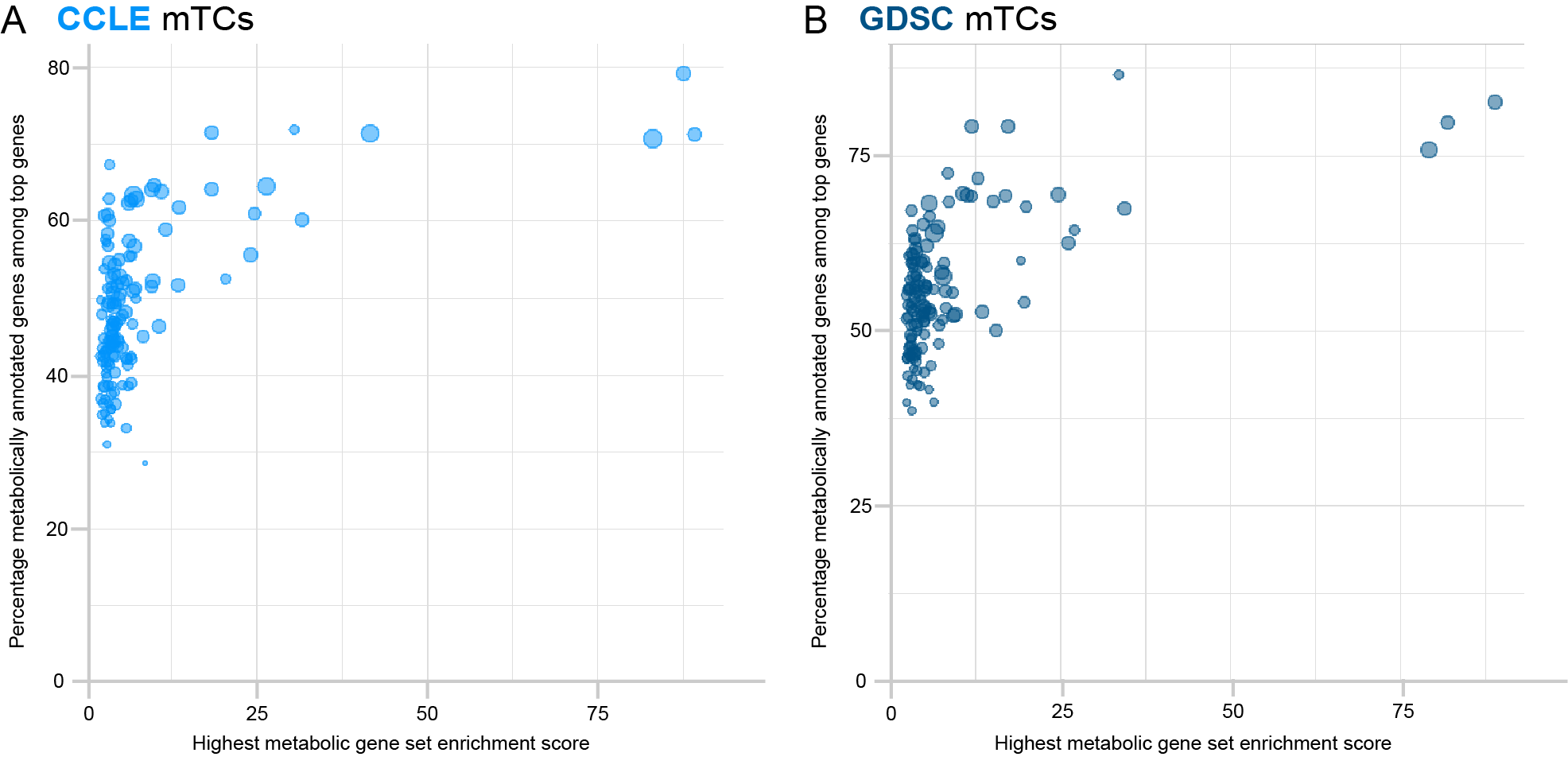
***

**Supplementary Fig. 5**

Dot plots showing the highest metabolic gene set enrichment score for every CCLE (A) and GDSC (B) mTC (x-axis) versus the percentage of metabolically annotated genes in the top genes (genes with absolute weight >3) in those mTCs (y-axis). The transparency of the dots is the same for all data points. Darker dot colors therefore mean that multiple data points are overlapping.

***
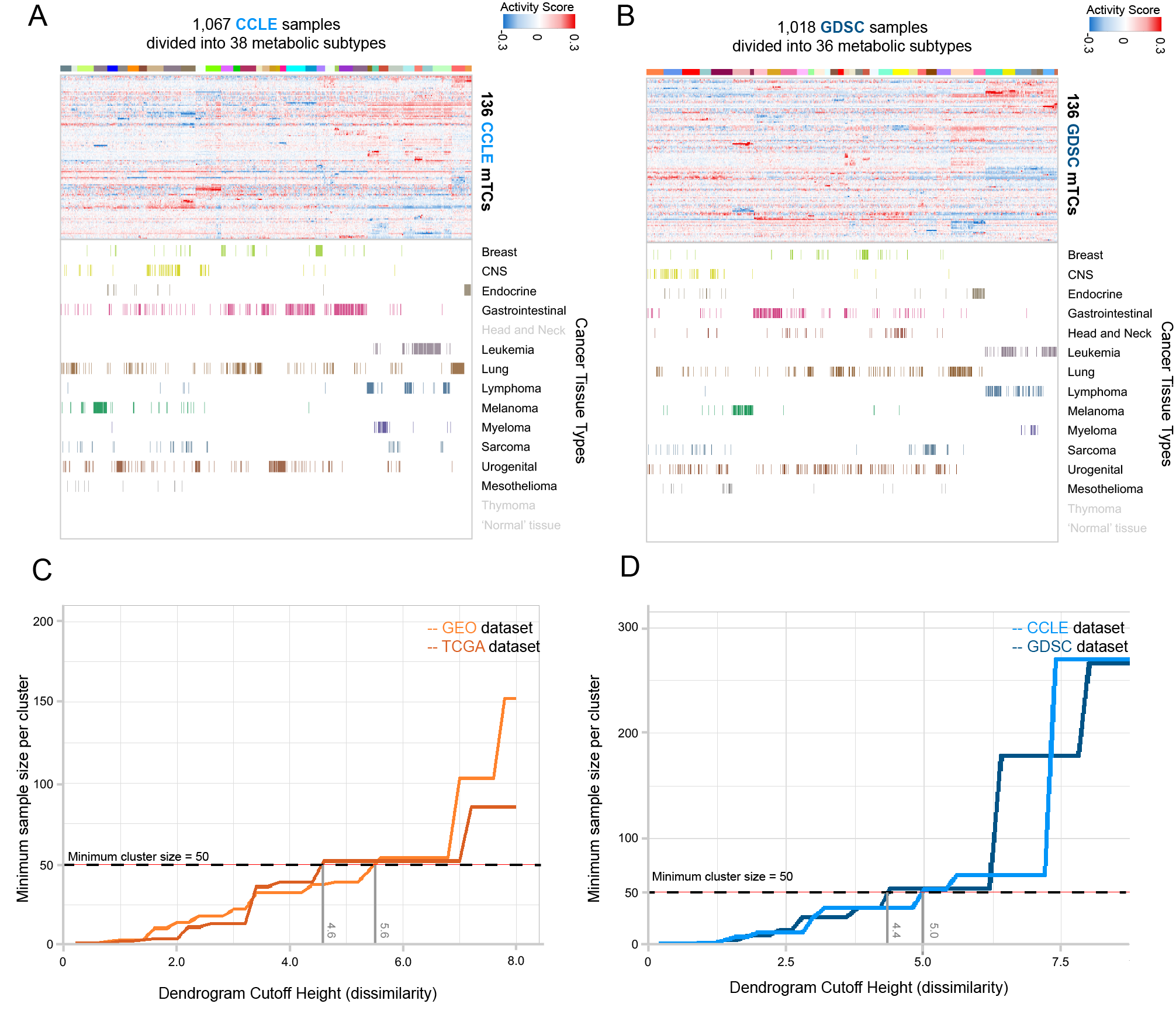
***

**Supplementary Fig. 6**

**(A)** Metabolic landscape for CCLE samples. The 1,067 samples were hierarchically clustered and divided into 38 metabolic subtypes. **(B)** Metabolic landscape for GDSC samples. The 1,018 samples were hierarchically clustered and divided into 36 clusters metabolic subtypes. Grey labels designate tissue types that are present in other datasets, but are not present in the given dataset.

**(C)** Hierarchical clustering of activity scores of mTCs in samples from GEO and TCGA datasets used in order to define metabolic subtypes. The plot shows the minimum sample size of a cluster depending on the chosen cutoff height of the dendrogram resulting from hierarchical clustering. The heights at which the minimum cluster size reaches 50 is given for both GEO and TCGA datasets. **(D)** Hierarchical clustering of activity scores of mTCs in samples from CCLE and GDSC datasets used in order to define metabolic subtypes. The plot shows the minimum sample size of a cluster depending on the chosen cutoff height of the dendrogram resulting from hierarchical clustering. The heights at which the minimum cluster size reaches 50 is given for both CCLE and GDSC datasets.


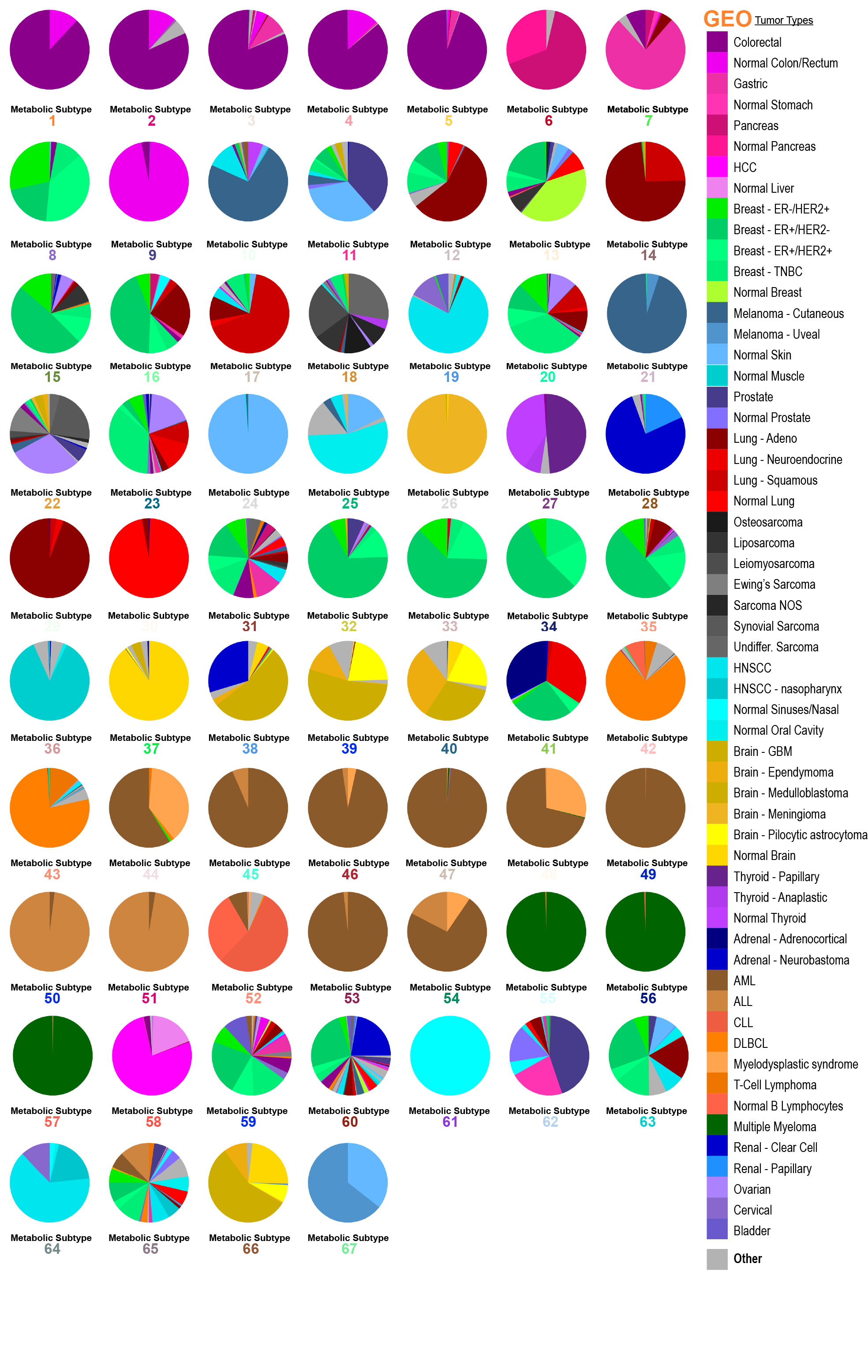


**Supplementary Fig. 7**

Pie graphs depicting the tissue type composition of the 67 metabolic subtypes defined for the GEO dataset.


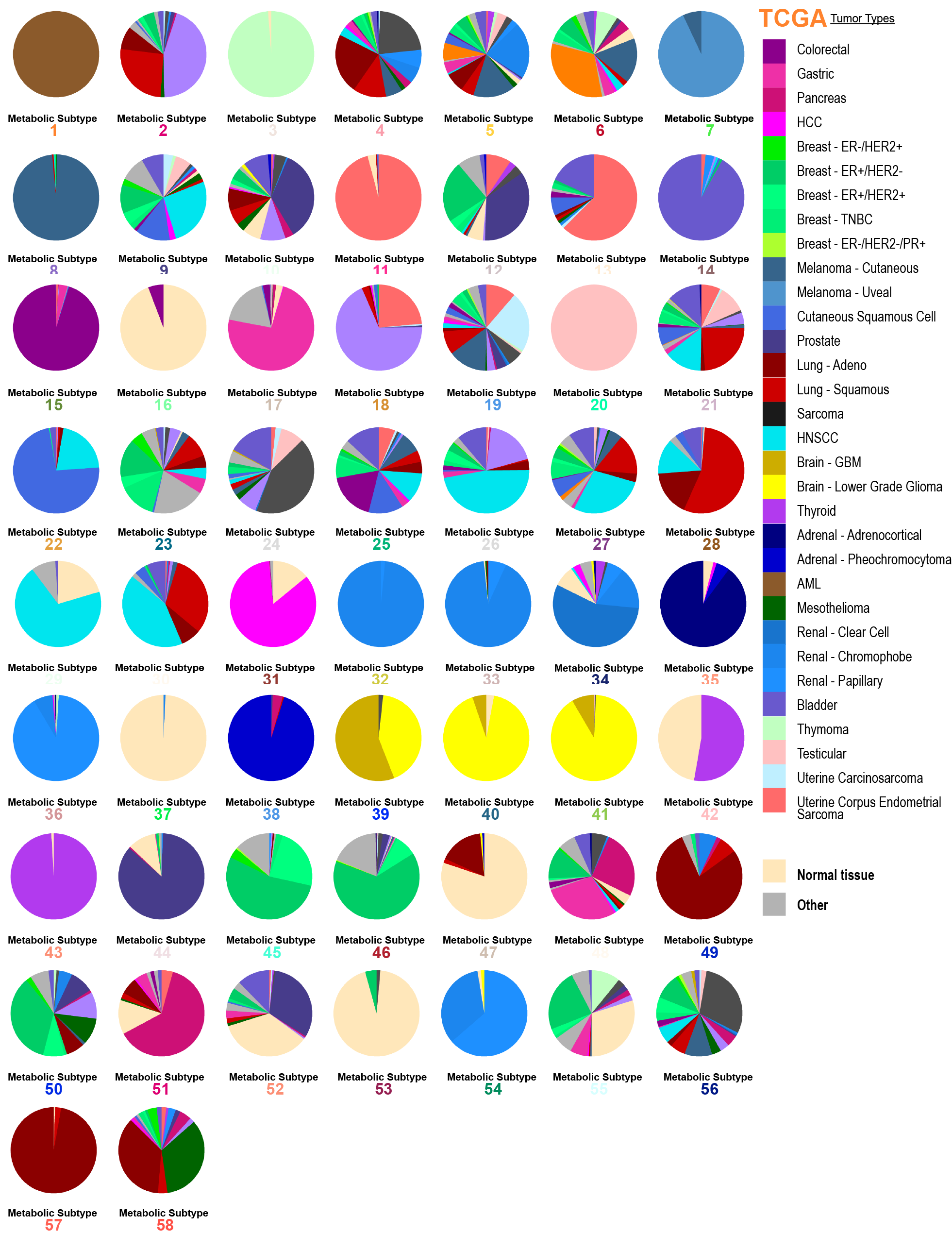


**Supplementary Fig. 8**

Pie graphs depicting the tissue type composition of the 58 metabolic subtypes defined for the TCGA dataset.


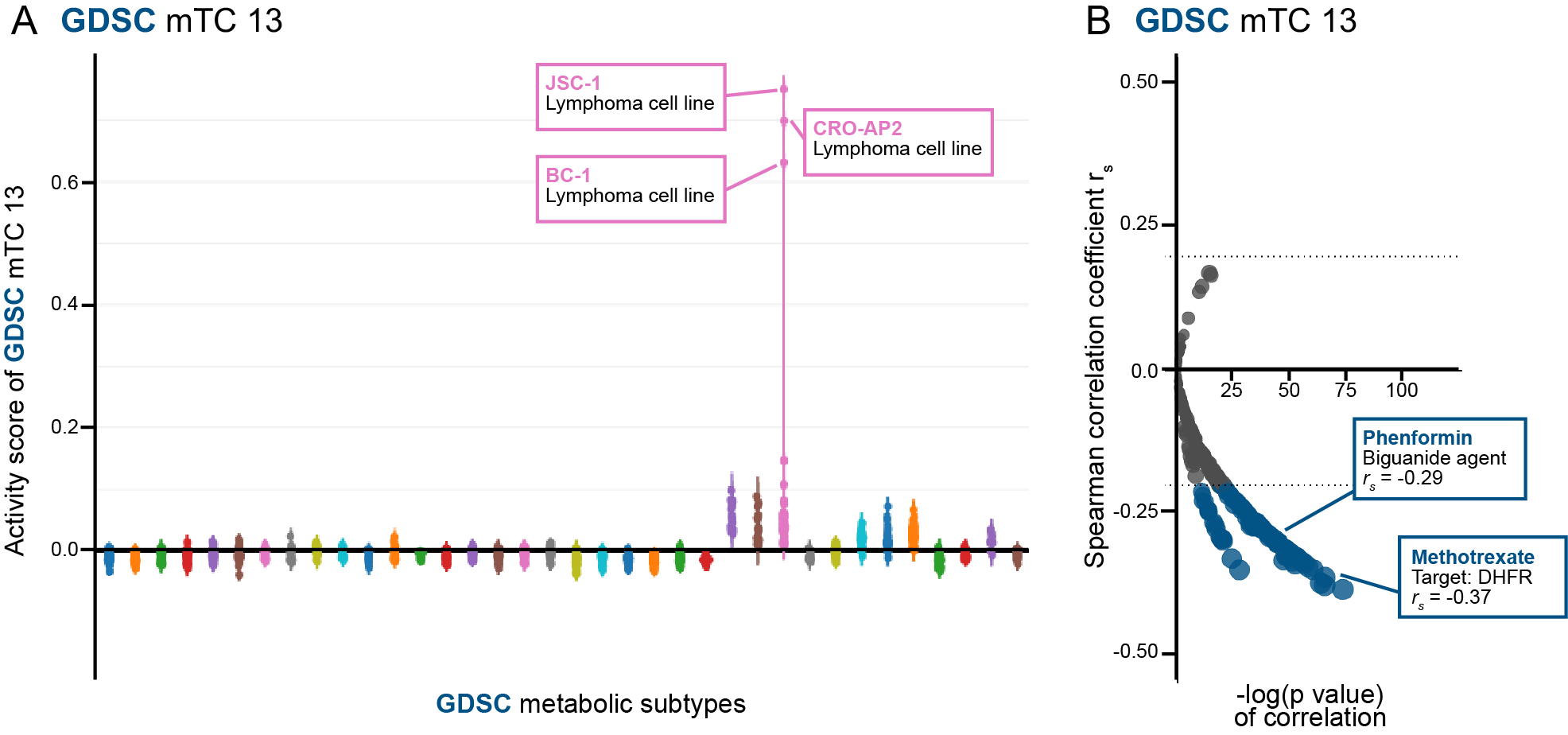


**Supplementary Fig. 9**

**(A)** Activity scores of GDSC mTC 13 in cell lines. **(B)** Spearman correlations between drug IC50 values and the activity of GDSC mTC 13.

**
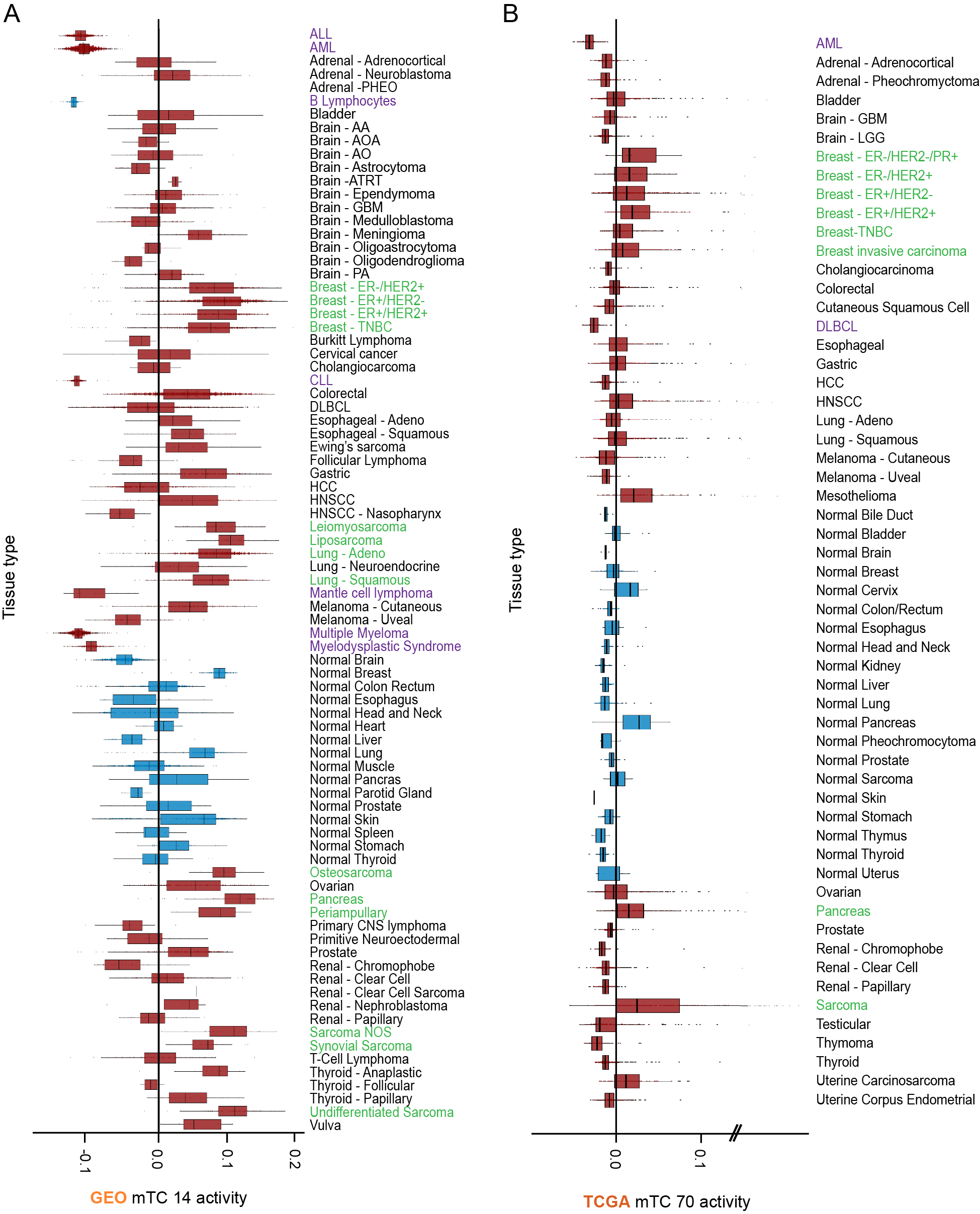
**

**Supplementary Fig. 10**

**(A)** Activity of GEO mTC 14 in samples, grouped per tissue type. Blue bars present normal tissue types, dark red bars represent cancer tissues. Tissue types with a higher median activity highlighted in the text are given a green axis label, tissue types with a lower median activity highlighted in the text are given a purple axis label. **(B)** The activity of TCGA mTC 70 in samples, grouped per tissue type. Blue bars present normal tissue types, dark red bars represent cancer tissues. Tissue types with a higher median activity highlighted in the text are given a green axis label, tissue types with a lower median activity highlighted in the text are given a purple axis label.

**
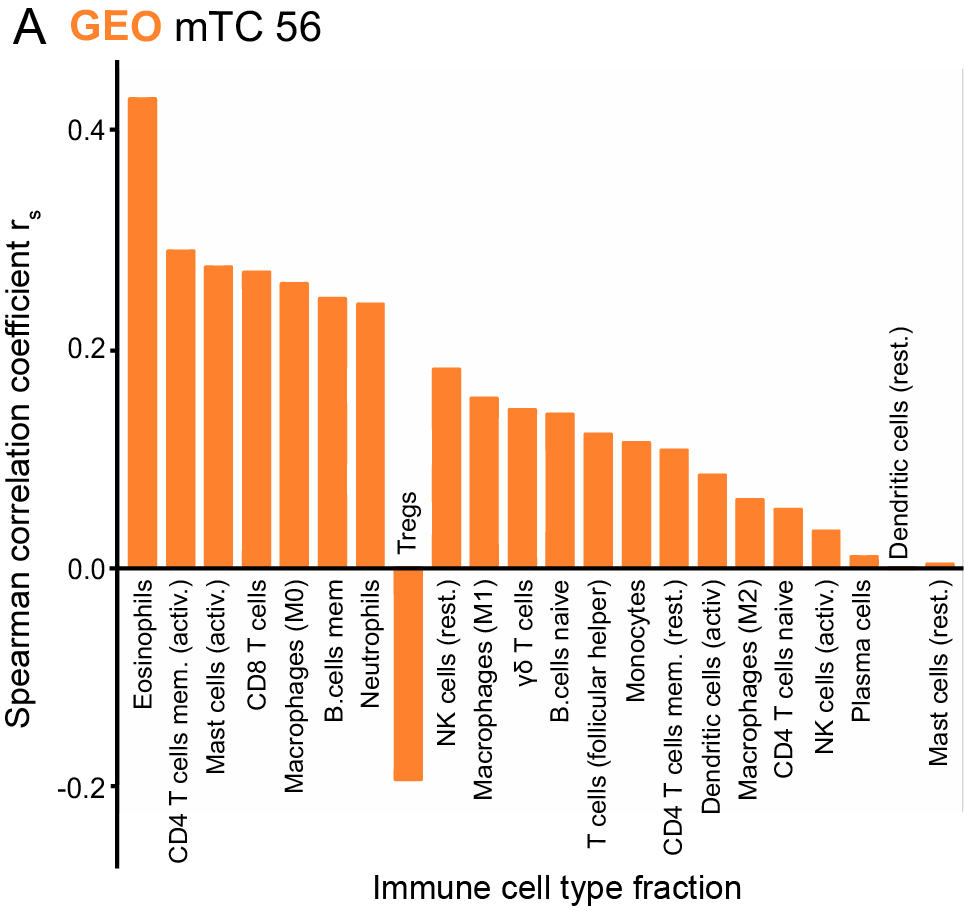
**

**Supplementary Fig. 11**

**(A)** Spearman correlations between CIBERSORT estimated immune cell fractions and the activity of GEO mTC 56.

**SUPPLEMENTARY METHODS**

***Data acquisition***

A detailed description of the data acquisition of the four datasets has been described previously (17). In short, the GEO dataset contained microarray expression data generated with Affymetrix HG-U133 Plus 2.0 (accession number GPL570). To select healthy or cancer tissue samples, a two-step search strategy was applied – automatic filtering on keywords followed by manual curation. Samples from cell lines, cultured human biopsies and animal-derived tissue were excluded. The TCGA dataset contained the pre-processed and normalized level 3 RNA-seq (version 2) data for 34 cancer datasets available at the Broad GDAC Firehose portal (https://gdac.broadinstitute.org/). The profiles in the CCLE dataset were obtained from the CCLE project, which conducted a detailed genetic characterization of a large panel of human cancer cell lines. Expression data within the CCLE project was generated with Affymetrix HG-U133 Plus 2.0. The GDSC dataset contained expression data generated with Affymetrix HG-U219. The aim of the GDSC project is to identify molecular features of cancer that predict response to anti-cancer drugs.

***Preprocessing, normalization and quality control***

A more detailed description has been provided previously (Bhattacharya et al., 2020). In short, preprocessing and aggregation of raw expression data (CEL files) within the GEO dataset, CCLE dataset and GDSC dataset was performed according to the robust multi-array average algorithm with RMAExpress (version 1.1.0). Quality control was performed on the GEO dataset, CCLE dataset and GDSC dataset separately with principal component analysis (PCA). Duplicate CEL files were removed by generating a message-digest algorithm 5 (MD5) hash for each CEL file. The expression levels for each probeset (in the GEO dataset, CCLE dataset and GDSC dataset) or gene (in the TCGA dataset) were standardized to a mean of zero and variance of one to remove probeset-specific or gene-specific variability in the datasets.

***Consensus independent component analysis***

We used consensus independent component analysis (c-ICA) to segregate the average gene expression patterns of complex biopsies into statistically independent transcriptomic footprints. The input gene expression dataset was preprocessed using whitening transformation, making all profiles uncorrelated and giving them a variance of one. Next, ICA was performed on the whitened dataset using the FastICA algorithm, resulting in the extraction of estimated sources (ESs) and a mixing matrix (MM). The number of principal components which captured 90% of the variance seen in the whitened dataset was chosen as the number of ESs to extract. Each ES contains all genes with a specific weight. This weight represents the direction and magnitude of the influence of an underlying transcriptional regulatory process on the expression level of that gene. The MM contains the coefficients of ESs in each sample, representing the activity of an ES in the corresponding sample. We performed 25 ICA runs with different random initialization weight factors to assess the robustness of the ESs and exclude ICA results derived from convergence at local solutions. ESs extracted from these runs were clustered together if the absolute value of the Pearson correlation between them was > 0.9. We calculated consensus transcriptional components (TCs) by taking the mean vector of weights in the co-clustering ESs. We considered a consensus TC robust when clustering included individual TCs from > 50% of the runs. The consensus TCs, in combination with the original input expression profiles, were used to obtain the consensus mixing matrix (MM) with the individual activity scores of the consensus TCs in each sample via matrix inversion.

***Identification of transcriptional components enriched for metabolic processes***

First, we selected gene sets defining metabolic process from five gene set collections obtained from the Molecular Signatures Database (MSigDb version 6.1); BioCarta, Gene Ontology – Biological Process (GO-BP), Gene Ontology – Molecular Function (GO-MF), KEGG, and Reactome. From BioCarta, gene sets were selected manually on the basis of their title. Selected gene sets described metabolic pathways or regulatory pathways regulated by metabolic processes. From GO-BP, all gene set were selected that contained the motif ‘METABOLIC_PROCESS’ in the title. In addition, gene sets that contained the name of a metabolite or class of metabolites in combination with the motif ‘_TRANSPORT’ in the title were selected. Furthermore, gene sets not containing these title motifs, but nevertheless associated with (cancer) metabolism, were manually selected on the basis of metabolic pathway names. From GO-MF, all gene set were selected that contained the name of a metabolite or class of metabolites in combination with the motif ‘_ACTIVITY’ or ‘_BINDING’ in the title. From KEGG, all gene set containing the motif “METABOLISM” or “BIOSYNTHESIS” in the title in combination with the name of a known metabolic route was selected. Furthermore, gene sets concerning metabolism-related regulatory pathways were selected on the basis of their titles. From Reactome, all gene set that falls within the hierarchy of the “Metabolism”-pathways were selected (see reactome.org/PathwayBrowser). The metabolism of Abacavir was not considered. A full list of all metabolic gene sets selected is presented in **Table S1**.

To identify transcriptional components enriched for metabolic processes, gene set enrichment analysis (GSEA) was performed using the selected metabolic gene sets. Enrichment of each metabolic gene set was tested according to the two-sample Welch’s t-test for unequal variance between the metabolic set of genes, which were under investigation, versus the set of genes that was not under investigation. To allow comparison between gene sets of different sizes, we transformed the Welch’s t statistic to a Z-score.

A biological process can be captured by multiple gene sets in several gene set collections. It is therefore possible that within the selection of 608 gene sets, multiple gene sets describe the same metabolic process. These will then show a similar pattern in gene set enrichment scores of transcriptional components. To reduce this redundancy, consensus clustering was performed gene set-wise on the GSEA data for the GEO, TCGA, CCLE, and GDSC datasets. Consensus clustering was performed using the ConsensusClusterPlus-package (v1.51.1) within R, using the default hierarchical clustering algorithm and Pearson correlation distance, a maximum amount of clusters (maxK) of 150, 2000 resamplings (reps), with 80% row and 80% column resampling (pFeature and pItem, respectively). The optimal number of clusters (*k)* was determined as the *k* at which the relative change in area under the CDF curve was minimized (<0.01). This resulted in a *k* of 50 clusters (**Figure S1**).

The 50 clusters of gene sets were subsequently used to select transcriptional components based on their enrichment for metabolic processes. Per gene set cluster, the three TCs with the highest absolute enrichment score for any gene set in that cluster were selected. In addition to this, the three TCs with the highest absolute mean enrichment score for all gene sets in that cluster were selected. The selected TCs were then referred to as metabolic Transcriptional Components (mTCs). In the end 4 different sets of mTCs were identified (GEO mTCs, TCGA mTCs, CCLE mTCs, GDSC mTCs)

***Pairwise gene level correlations of mTCs between datasets***

To correlate two mTCs of different datasets, first the subset of genes that had an absolute weight higher than 3 in two mTCs were selected. Then, the overlap between these two sets of top genes was determined. Using the gene weights of the overlapping genes in both mTCs, pairwise correlations were calculated. Specifically, Spearman correlations were performed in R using the *pspearman*-package (v0.3-0) in R, with a t-distribution as approximation to determine the p-value. As the amount of genes with an absolute weight above 3 was different for every mTC, the size of the overlap in genes between two mTCs changed as well. The significance of the Spearman correlation found between two mTCs therefore was dependent on the number of overlapping genes. Hence, additionally the significance of the found size of the overlap in genes between mTCs should be determined. To this end, for a pair of mTCs, two sets of random gene identifiers were selected from all possible gene identifiers. The amount of randomly selected genes per set corresponded to the number of genes with a weight >3 in both mTCs. Subsequently the overlap in gene identifiers between the two random sets of gene identifiers was determined. By repeating this 10,000 times, the chance of finding a given overlap between two sets of genes could be determined.

Ultimately, mTCs were said to be concordant when their correlation was > 0.5, with a P value < 0.05, given that there was a significant overlap in genes (P value of overlap <0.05).

***Clustering of Metabolic Transcriptional Components, Genes and Samples***

For each of the four datasets, the matrix containing gene weights for every mTC was clustered on genes. To this end, hierarchical clustering was performed using ward.D2 as the method and 1-cor(data) as the distance function. Heatmaps were created using R’s *gplots* package (v3.0.1).

For each of the four datasets, the mixing matrix (MM) containing activity scores were clustered both on samples as well as mTCs. To this end, hierarchical clustering was performed using ward.D2 as the method and 1-cor(data) as the distance function. Heatmaps were created using R’s *gplots* package (v3.0.1). On the basis of the MM clustering for every dataset, metabolic subtypes were defined. To determine the sizes of clusters of samples that would make up a metabolic subtype, the dendrograms resulting from hierarchical clustering of the samples was systematically cut at dissimilarity values ranging from 0.0 to 8.0 with increments of 0.2. For each of the four datasets GEO, TCGA, CCLE and GDSC, the cutoff was chosen at such a dendrogram height at which the smallest cluster reached a size of 50 samples (**Figure S6**).

***PAM50 molecular subtyping***

All GEO samples annotated as breast cancer were PAM50-subtyped using the *genefu* package (v2.24.2) which is available through Bioconductor. To this end, the pre-processed and normalized expression profiles as generated with the Affymetrix HG-U133 Plus 2.0 (accession number GPL570) were used. The *molecular.subtyping()* function was used with the argument *do.mapping=TRUE* to perform mapping through Entrez Gene ids.

***CIBERSORT***

Relative and absolute immune fractions for 22 immune cell types were estimated for all samples in GEO and TCGA datasets using the CIBERSORT algorithm running with default parameters, 1000 permutations and selecting ‘nosumto1’ as output. This output was then associated with the activity of the mTCs in samples, through spearman correlation.

***Statistical Analyses***

Univariate OS on breast cancer samples from GEO and univariate DRFS analyses on melanoma samples from TCGA were performed using a cox regression model through *survminer* (v0.4.3) and *survival* (v2.43-3) packages in R. Confidence intervals were set at 0.95, and significance was tested through the Log Rank test. Scripts are available at github.com/**MetabolicLandscape/.** Pearson correlations were performed in R using the cor.test()-function from the *stats* package (v.3.5.1). Spearman correlations and the corresponding exact p-values were calculated using the *pspearman*-package (v0.3-0) in R, with a t-distribution as approximation.

**Additional resources**

All identified mTCs and additional analyses can be explored at http://themetaboliclandscapeofcancer.com.
